# Composite Electrolytes for Non-Lithium-Ion Batteries †

**DOI:** 10.3390/polym17223084

**Published:** 2025-11-20

**Authors:** Qunting Qu, Lili Liu, Lijun Fu, Xuecheng Chen, Yuping Wu, Rudolf Holze

**Affiliations:** 1 College of Energy, Soochow University, Suzhou 215006, China; 2 State Key Laboratory of Materials-Oriented Chemical Engineering, School of Energy Science and Engineering, Nanjing Tech University, Nanjing 211816, China; 3 Faculty of Chemical Technology and Engineering, West Pomeranian University of Technology in Szczecin, Piastow Ave. 45, 71-311 Szczecin, Poland; 4 Confucius Energy Storage Lab, School of Energy and Environment & Z Energy Storage Center, Southeast University, Nanjing 211189, China; 5 Department of Electrochemistry, Institute of Chemistry, Saint Petersburg State University, 7/9 Universitetskaya Nab., 199034 St. Petersburg, Russia; 6 Chemnitz University of Technology, D-09107 Chemnitz, Germany

**Keywords:** non-lithium batteries, zinc-ion battery, magnesium-ion battery, sodium-ion battery, potassium-ion battery, aluminum-ion battery, electrolytes, electrolyte solutions, solid electrolytes, polymer electrolytes, composites, filler, hybrid materials

## Abstract

Composite electrolytes for applications in batteries and supercapacitors, i.e., in electrochemical energy technology, are gaining growing attention. In the absence of a commonly accepted definition, binary and ternary combinations of materials, e.g., a polymer with an electrolyte salt or electrolyte salt solution and a third conductivity- and performance-enhancing constituent, are assumed as a definition of a composite electrolyte in the following review. Relevant fundamentals and reported research results, including explanations of the described effects of added ingredients and achieved improvements, are reviewed. Future perspectives and directions of further research are sketched.

## 1. Introduction

The ionically conducting phase between the two electrodes of a supercapacitor or a battery is frequently and somewhat imprecisely called the electrolyte. According to the commonly accepted definition, an electrolyte is composed of ions (a true electrolyte according to standard textbooks [1], e.g., NaCl) or of molecules, which dissociate into ions upon interaction with a suitable solvent (so-called potential electrolytes, e.g., HCl in water), and accordingly, in most cases, electrolyte solutions or molten electrolytes (salts) are employed [2,3,4]. Ionic liquids (ILs; in this report, commonly established acronyms [5] are used independently of some authors’ different, but confusing, suggestions) composed of ions only and liquid already at room temperature (room-temperature ionic liquids RTIL) are the rare example in the latter group. In the following text, this distinction between electrolyte and electrolyte solution is ignored for the sake of simplicity and convenience, but mostly because solutions play hardly any role at all, except in the preparation.

Initially, because of safety concerns and risks associated with leakage of devices, the use of liquid electrolytes (solution) was not welcome in most applications [6]. Consequently, numerous means to replace them with non-liquid, semi-solid, or even solid ion-conducting materials have been proposed and examined; for an overview of recent driving forces, trends, and new opportunities, see [7]. These attempts started with a general “wish list” for an electrolyte system [6,8]:

- Wide available electrode potential window or window of electrochemical stability;
- High ionic conductivity at common operating temperatures;
- Sufficient chemical and electrochemical stability;
- Compatibility with electrode and separator materials;
- Thermal stability;
- Environmental compatibility;
- Low price;
- Sustainable resources.

Given the stated flaws, limitations, and challenges of liquid electrolytes, attempts to improve on those via particular solid electrolytes have implicitly or explicitly addressed the following approaches:

- Enhanced ionic conductivity;
- Wider range of operating temperatures;
- Improved mechanical stability;
- Better long-term stability;
- Increased thermal stability.

The crucial and persistent problem of establishing and maintaining electrolyte/electrode interfaces between solid electrodes and solid electrolytes is surprisingly addressed only infrequently; for overviews, see, e.g., [8,9].

Initially single-constituent solid electrolytes were studied mostly for high-temperature applications in, e.g., sodium/sulfur or sodium/metal chloride batteries. Frequently encountered/employed solid ion conductors for this application and beyond are as follows [10]:

- Perovskites, e.g., (Li,La)TiO_3_;
- Garnet-like Li_5_La_3_M_2_O_12_ (with M = transition metal);
- Mostly amorphous glasses of lithium nitrides, sulfides, borates, or phosphates like lithium phosphoroxydnitride (LiPON);
- “Super ion conductors” of the LISICON or NASICON type: Li(Na)M_2_(PO_4_)_3_ (M = Ti(IV), Zr(IV), Ge(IV));
- Lithium salts like LiI in Li/I_2_ batteries [11].

Unfortunately, these materials frequently show inadequate ionic conductivity; in addition, problems in difficult processing and slow deterioration because of, e.g., phase changes limit their suitability. Combining them with further ingredients into composite materials was identified as a promising option. A very early report by Liang [12] from 1973 provided convincing evidence that the addition of around 45 mol% of Al_2_O_3_ to LiI resulted in a very significant increase in ionic conduction to around σ_RT_~10^−5^ S/cm. Solid-state batteries—in particular, high-temperature batteries—of the type Li/LiI(Al_2_O_3_)/metal iodide were prepared and tested [13]. Electronic conductivity of the created composite electrolyte was negligible; the dominant ionic conduction mechanism was proposed to proceed along cation vacancies. Similar observations with other electrolytes, e.g., AgI with Al_2_O_3_ [14] or LiI with SiO_2_ [15], have been reported later. An understanding of the conductivity enhancement could not be reached. Acceleration of charge carrier movement along the interfaces between electrolyte and filler was suggested [16]. First hints suggested space-charge-layer formation and a contribution to accelerated ion transport in LiBr hydrate [17]; surprisingly, earlier, these considerations were elaborated in great detail for the electrolyte CuCl_2_/Al_2_O_3_ [18]. In later studies, this increase has been attributed to the creation of space charges at the filler/matrix interface, in particular, by surface-active but otherwise inert additives [19]. Another interpretation and explanation based on lattice considerations and Monte Carlo simulations appears to be less helpful when studying polymer/filler composites [20]. Effects of Lewis acid/Lewis base interactions at said interfaces and with anions and/or cations of the mobile charge carriers have been considered, and they may be of particular interest in understanding changes in transference numbers caused by, e.g., selective adsorption of only one kind of mobile ion [21]. Actually, initial observations of enhanced ionic conductivity caused by admixing a second component into an ionic crystal date back to Jander [22]. Only recently, the special role of filler–bulk interfaces and the conditions influencing ion transport therein has been transformed into the creation of continuous interfaces in the bulk of a solid ion conductor, yielding ion conductors with remarkably high conductivities [23]. Mechanochemical synthesis methods of solid electrolytes have been reviewed [24].

With the discovery of various polymeric materials also suitable as ionic (or ion) conductors, in particular, after the addition of electrolytes, i.e., salts (for the earliest example, see [25]), the question was asked again about the cause of increased conductivity upon the addition of a filler to a polymer with added salt. Early observations indicated no enhancing effect at, e.g., less than 10 wt.% Al_2_O_3_ to poly(ethylene oxide) (PEO; for details, see below) with LiClO_4_ as the salt [26]. In any case, crystallinity of the polymer was identified as a major cause of poor conductivity, and lower crystallinity increased conductivity—thus, added materials that decrease crystallinity are welcome fillers. For polymer-based solid electrolytes, for some time, the crystallinity argument pushed the space charge argument promoted for inorganic crystalline conductors completely into the shadows. Only later was this contribution rediscovered for polymeric electrolytes together with further effects related to chemical interactions between the filler surface and the molecular structure of the polymers and the ions of the electrolyte commonly added to the polymers [27,28,29,30].

Up to this point, the common definition of a composite as a material wherein two phases can be discerned at least on the microscopic level, not necessarily on the macroscopic one, was tacitly and unreflectedly applied. Solutions of several salts in a solution or addition of one more solute did not and do not make a composite. Depending on the type and extent/intensity of interactions between the constituents, the more general term “composite” may be correctly replaced with the more focused term “hybrid”. Because there is no generally accepted and clear-cut separation between both terms, the usage by the original authors is followed in this contribution without discussing the adequacy of this terminology in every case; an extended review on “hybrid electrolytes” is available [31]. Actually, the phase boundaries established between the surface of filler nanoparticles and polymeric matrices frequently provide extra fast pathways for ion transport—this may be called a typical case of an interaction calling for the term hybrid, as in [32], whereas it remains somewhat confusing to call a mixture of salts and liquids simply a “hybrid electrolyte” [33] or to call just everything beyond a solid electrolyte a “hybrid electrolyte”, as in [34]. Certainly, it is wrong to attribute this—as is the case in [34]—to the authors of [35]: the claimed figure is nowhere to be found in the latter report. Clearly, this is a minority opinion anyway; nevertheless, the search string “(“hybrid electrolyte” OR “solid electrolyte”) AND (sodium OR magnesium OR potassium OR zinc OR aluminium OR calcium) AND battery” was tried in our literature searches. These interactions have been reviewed with particular attention to NMR as an experimental tool in a previous study [36].

Another option for increased conductivity is the use of additives: If a small amount of another constituent is added, the result may also be called a composite; for examples, see [37]. Similar imprecision can be found when an electrolyte solution containing several electrolytes is called a composite electrolyte, for examples, see [38,39,40,41,42,43,44,45,46,47,48,49,50,51], or, with again an additive, a “solvation structure composite electrolyte” in [52]. “Mixture” is certainly correct and more appropriate, though unfortunately less fashionable.

Essentially, the same arguments prevailed when interest in solid electrolytes as replacements for liquid electrolyte solutions in lithium-ion batteries almost exploded, stimulated at least in part by spectacular battery failures [53]. But similar limitations were also readily registered [54]. The same approaches toward improvement were tried: Composite materials are one option.

Finally, the limitations of lithium-ion battery technology and its economy, despite its many impressive advantages, stimulated research into alternatives, so-called post-lithium or non-lithium batteries. Although it appears that both terms are sometimes almost synonyms, they need proper usage: Post-lithium commonly means everything beyond the omnipresent lithium-ion battery based on the rocking-chair principle and thus can include lithium–air and lithium–sulfur [55], whereas the term non-lithium is much better defined—and applied here. Composite electrolytes for the former lithium-ion batteries are the subject of a review elsewhere [56]; materials for the latter, much wider but less densely populated family are the subject of this report. Earlier reviews covering a few details of the present contribution are available [57,58].

**Figure 1:**
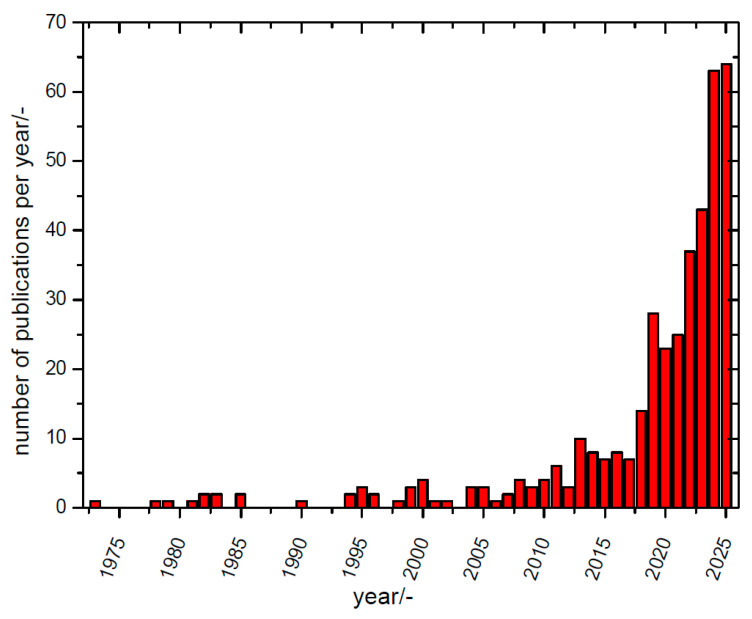
A search with the string ““composite electrolyte” AND (battery OR batteries) NOT lithium” found anywhere in the title, keywords, or abstract (data from Scopus^®^ and Web of Science^®^) performed on 2 August 2025 initially yielded 304 hits. Reports about, e.g., lithium-ion conductors with the term “lithium” somewhere in the text but not in the title, keywords, or abstract were removed, even those on Li. Reports dealing with electrolytes actually meeting the definition suggested above without mentioning the term “composite electrolyte” were included when detected after extended literature searches. Cases where (composite) “separator” was considered a synonym of (composite) “electrolyte” are also included (for a typical example, see [62]), and cases where a “composite separator” was actually a modified separator only (for examples, see [63,64,65,66,67,68,69,70,71,72,73]; for a review, see [74]) were not included. Modified membranes considered as separators and called composites like [75,76] are not included; neither are reports dealing with “moderate electrolytes” [77] nor electrolyte solutions of salt mixtures [38,39,40,41,42,43,44,45,46,47,48,49,50,51,78], solid electrolyte interphases (SEIs), or similar electrode coatings, as in [49,51,79,80,81,82,83,84,85] or reports wherein “composite electrolyte” is placed only as a teaser in the abstract [86,87]. This changed the total to 394 reports.

Early reports on composite materials, mostly on solid/solid composites, as solid electrolytes for batteries were already collected in a conference proceedings volume in 1988 [59], followed by a conference report on a composite material used as a ceramic solid-state electrolyte for a sodium–sulfur battery (1990) [60], and a report in 1995 on solid-state batteries with a negative magnesium electrode and various compounds as the positive electrode, with a solid composite electrolyte as a glue in between [61]. But only in the 2010 years was there growing general interest in non-lithium battery research, and accordingly, publication activity picked up, as depicted in Figure 1.

Although the majority of composite electrolytes are solids—in line with the general ambitions of working towards all-solid-state batteries—liquid electrolytes avoiding the problem of flammability are still studied. Eutectic electrolytes based on experiences with deep eutectic solvents [88,89,90,91,92,93,94,95,96] are a prominent example, which have been reviewed [97]. Fibrous materials as scaffolds for composite electrolytes/membranes/separators have also been surveyed [98]. Lignocellulose as a typical renewable raw material has been discussed with respect to applications in batteries and composite electrolytes [99], and respective overviews on the use of nanocellulose [100] and cellulose [101] are also available. Composites of metal–organic frameworks as part of solid electrolytes have been reviewed [102].

The following report collects the most relevant details of original reports on composite electrolytes studied or at least proposed for the currently researched non-lithium batteries. It is organized along these metals, and in cases of frequently studied metals, there are subsections using the mostly polymeric host material as a criterion. This should provide the reader with a quick overview of previously examined options and materials and serve to identify promising avenues of further studies.

## 2. Electrolyte Tasks and Challenges

In conventional electrolytes as well as electrolyte solutions, the roles played by the ingredients are perfectly clear in almost every case [2,3,4,5,6,7,8,9,10,11,12,13,14,15,16,17,18,19,20,21,22,23,24,25,26,27,28,29,30,31,32,33,34,103]. Once a further ingredient is added into an electrolyte, e.g., a further ionic or non-ionic compound to a salt, there should be a reason specified together with the suggestion of the addition. Unfortunately, as in many examples presented below, this is not always be the case, and the reader is left wondering about conceivable ideas behind the addition. Depending on the battery chemistry and the operating principle, high general ionic conductivities and, in particular, high conductivities (i.e., high transference number *t*_+_ ≈ 1) of the (mostly metal) ion shuttling between the electrodes are highly welcome. This selectivity may also be helpful by suppressing cation concentration gradients and thus suppress dendrite formation when using metal (e.g., lithium, sodium) electrodes. When authors address this detail, it is highlighted below.

### 2.1. Composite Electrolytes

**Figure 2:**
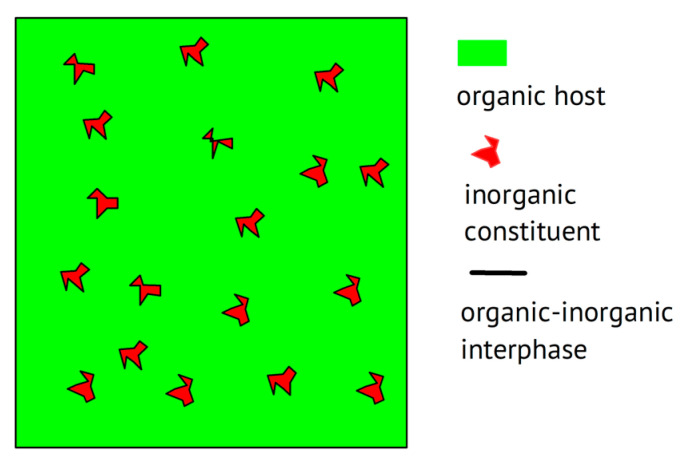
Schematic of a composite with a polymer host.

Beyond the trivial statement that any combination of two materials enabling ionic conduction without showing electronic conduction—this may even qualify as the definition of an electrolyte—may be called a composite (wherein these two constituents stay separate in two phases at least on the microscopic level, as illustrated below in Figure 2), the goal of combining materials is commonly pursued with particular improvements in mind. These may range from clear improvements in terms of the electrochemical properties listed above in the wish list to further technical and even economical improvements. When examining composites with a polymer, the mechanical scaffold provided by the macromolecular material—serving as a welcome stiff material rendering a separator possibly superfluous—combined with an improvement of the ionic conductivity of the polymer the focuses of attention. In the case of composites composed of two or more non-polymeric materials, the welcome mechanical contribution of a polymeric scaffold remains absent; another advantage beyond an improved ionic conductance will be the driving force behind mixing. Use of the sometimes-encountered term “filler” for the added ingredient (see above) appears to be a bit unclear, or at least undefined. The distinction between passive (insulating) fillers not providing internal ionic conduction and active (conducting) fillers suggested in [104,105] has apparently not found widespread use. To call the salt added to a polymer to provide ions a “filler” appears to be positively confusing [106]; neither makes the term “composite electrolyte” reasonable for a combination of an insulating polymer with a salt, as in [107].

#### 2.1.1. Composites with Polymer Hosts

Overviews on polymer electrolytes are available [108,109,110,111,112]. Starting a composite electrolyte with a polymer or a polymer electrolyte is very rational: The polymer will provide the mechanical support (or backbone, but this should not be confused with the chemical structure or molecular backbone) to form a scaffold, desired for an application not needing an extra separator. The added (composite) constituent may, e.g., reduce the crystallinity of a polymer like PEO, thus increasing ionic conductivity [113,114], or may provide particularly transport-accelerating particle/host interfaces, as demonstrated in [30], essentially along the lines already indicated in [19]. A schematic representation is shown in Figure 2, highlighting these interfacial or interphase regions.

**Figure 3:**
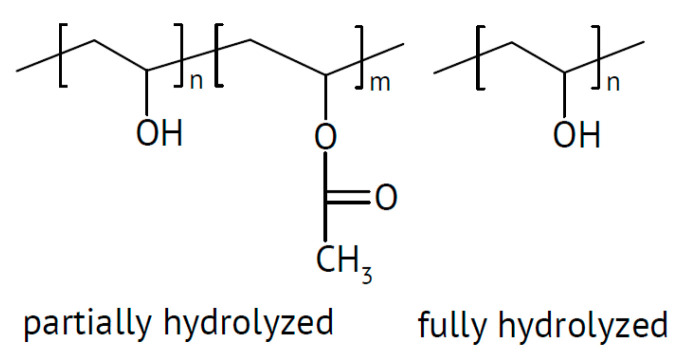
Molecular structures of poly(vinyl alcohol).

**Figure 4:**
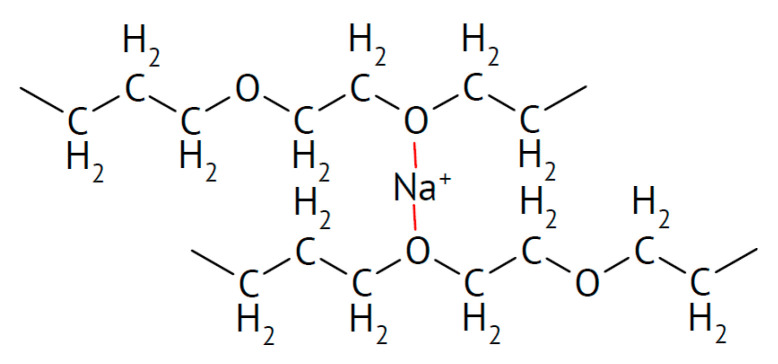
PEO and a scheme of interactions **----** between a sodium ion and PEO.

**Figure 5:**
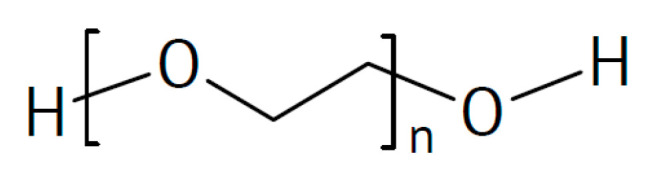
Molecular structures of poly(ethylene glycol).

**Figure 6:**
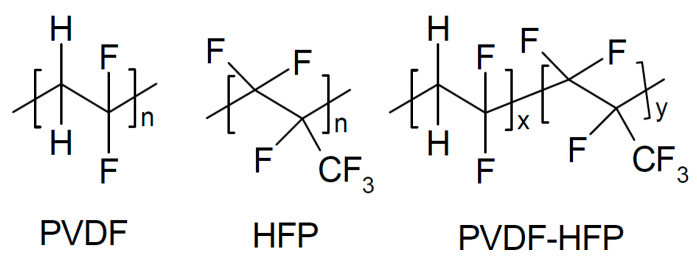
PVDF, HFP, and PVDF-HFP (this acronym is chemically slightly incorrect but is—with very few (see below) misleading exceptions—firmly established and thus used here) [120].

There is also evidence that effects may depend on the particle size, with small particles increasing conductivity only and larger ones increasing the conductivity and transport number [115]. This applies to all added particular materials. In a further highly unsystematic study of several inorganic components with different particle sizes and dielectric constants, no coherent conclusions were obtained [116]. Ion-conducting materials may add further ionic conduction pathways inside the particles. Possibilities of enhanced ionic conductivity mechanisms have been reviewed [117]. Further advantages may be gained by starting from a polymer blend of, e.g., poly(vinylalcohol) PVA (Figure 3) and PEO (Figure 4). Beneficial effects were also observed upon the addition of BaTiO_3_ as a filler to a polymer blend of PVA and poly(ethylene glycol) PEG (Figure 5), of Li_0.5_La_0.5_TiO_3_ to a blend of PEO and polyvinylidene fluoride (PVDF, see Figure 6), or of ZrO_2_ to poly(vinylidene fluoride-co-hexafluoropropylene) (PVDF-HFP, see Figure 6). Actual modes of operation of the added ceramic may differ from material to material depending on the exact chemical composition. The case of PEO with inorganic fillers has been reviewed in detail [118]. In addition, attention has been paid to effects of surface modifications of inorganic fillers added to PEO [119].

Some general procedural aspects of polymer and composite polymer electrolytes (preparation and handling) have been summarized in [121]. Tuning of the interface between the polymer matrix and embedded mostly inorganic particles (filler) in a solid electrolyte, preferably for photovoltaic devices, has been reviewed [122]. (Gel) Electrolytes obtained by plastification (gelling) of a polymer with a suitable solvent (this may be a solvent or a solvent mixture but also an electrolyte solution) have been called composite electrolytes infrequently (for an example, see [123]). Addition of a plasticizer to a polymer appears to be so popular that it is hardly mentioned in the reports, and the same applies to the essential addition of a source of ions, e.g., a sodium salt, either during the preparation of the solid or by soaking the prepared solid in an electrolyte solution—wherein the solvent may also act as a plasticizer. Again, these are details the interested reader should search for in an attempt to avoid experimental failures.

An overview covering PEO-based composite electrolytes with a major focus on batteries is available [124], and composites with Li_7_La_3_Zr_2_O_12_ as an inorganic filler have been discussed in [125]. Beneficial effects of added small molecules in a polymer–ceramic solid electrolyte are discussed in general terms in [126]. Segmental motion of the polymer and interfacial polarization are coupled and determine ion transport jointly. A porous inorganic scaffold of freeze-cast Li_7_La_3_Zr_2_O_12_ for filling with a polymer yielding a composite has been reported [127]. Further polymer–inorganic filler composites have been reviewed with respect to mass production of solid-state batteries [128], and early overviews on polymer–ceramic electrolytes already highlighting the three overarching issues ionic conductivity, transference number, and interfacial aspects are available [129,130]. Fast and efficient discovery of solid organic–inorganic composite electrolytes supported by unsupervised learning has been discussed in detail [131]. Approaches to reaching an optimized inorganic filler content enabling better electrolyte–electrode contact supported by machine learning have been discussed [132]. The beneficial, ion conductivity-increasing effect of nanosized particles of TiO_2_ added to amorphous polyether with either LiClO_4_ or LiTFSI as a salt has been studied [133]. At most studied concentrations, no effect was observed; only at 1.5 m LiClO_4_ was an increase observed and attributed (somewhat surprisingly) to an effect on crystallinity and disruption of ion aggregation.

Further general aspects of polymer-based composite electrolytes have been discussed [134,135,136].

#### 2.1.2. Binary Composites

**Figure 7:**
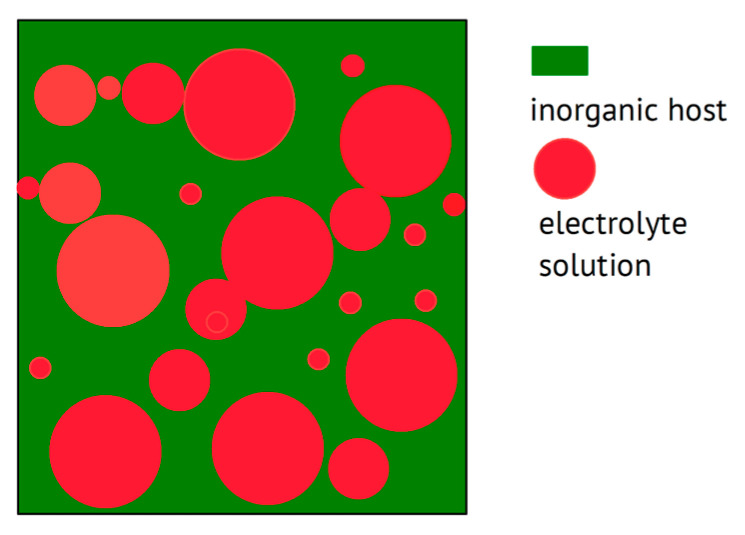
Scheme of a porous inorganic host filled with an electrolyte solution.

Instead of a polymer acting as a host and providing in most cases the major fraction of a composite electrolyte, an inorganic material like fumed silica may act as a highly porous host material, which is subsequently soaked with an electrolyte solution, yielding a solid material (for the concept of the “soggy sand” electrolyte, see [137,138]), which may not possess the mechanical properties enabling its use as a separator or similar; for a scheme, see Figure 7.

Ultrafast high-temperature sintering as a method for preparing an inorganic scaffold for a composite electrolyte has been described [139].

Given the wide variations in terms of employed compounds and constituents as well as battery chemistries, organization of the material making it easily accessible for the reader was based on a rough discrimination between room-temperature (RT) and elevated-temperature applications, with a further classification according to the metal used in a given system for the RT batteries and a further distinction based on the polymer host material.

The presumably central challenge of a sufficiently intimate contact between electrolyte and electrode—in particular, positive ones—is somewhat surprisingly addressed only sometimes, while the traditional sandwiching of electrodes with an electrolyte in between is still popular. Because this interface has been identified as a major problem [8,54], the following attempts to enhance this interface are highlighted.

The most prominent property of an ionic conductor is its ionic conductivity. Accordingly, measured data attract the most attention. For a given application, the actual Ohmic resistance caused by the ionic phase between the electrodes is much more relevant—but rarely reported, only sometimes included in some cell resistance and cell impedance data. A highly conducting material that can be applied only as a relatively thick layer may thus be less attractive than a relatively poorer conducting material that can be made into a very thin film. Nevertheless, data—when reported—are included below to provide at least a first number. Given the pronounced temperature dependency of ionic conductivity, most authors provide values—if at all—for a specified temperature, like room temperature σ_RT_ or σ_60°C_; when this basic detail is missing, σ_?_ is provided. Temperatures like *T* = 50 or 60 °C are still found in the part of this report dealing with room-temperature systems because the authors do not explicitly mention the use of the reported materials at elevated or high temperatures. Some general information on experimental testing aspects can be found [140], and some early materials not assigned to a particular battery chemistry have been described [141]. The use of NMR and EPR spectroscopy for in situ studies of rechargeable batteries has been reviewed [142].

## 3. The Materials

### 3.1. Room-Temperature Systems

The careful distinction between lithium and lithium-ion or more generally metal and metal-ion batteries with the rocking-chair principle operative in the latter ones may be applicable also for post-lithium batteries. Evidence suggests otherwise and confusion reigns, and the following assignments picked by the authors were used when specified at all. These include the explicit use of the term “metal-free” (like sodium-metal-free in [143]), which so far appears to be reserved for batteries where essentially a negative metal electrode formed only by metal deposition from the electrolyte solution/dissolution without the solid metal being present at the start (enabling simplified manufacturing) is used (see for examples, [144,145,146,147,148]) and not for a cell with a host electrode. Actually, even these cells are not called metal-free (which appears to be a rather striking characterization anyway) but “zero excess metal”, “reservoir-free”, or “anode-free” (with the precision in describing the facts decreasing in this sequence).

#### 3.1.1. Sodium and Sodium-Ion Batteries

##### Inorganic Materials

**Figure 8:**
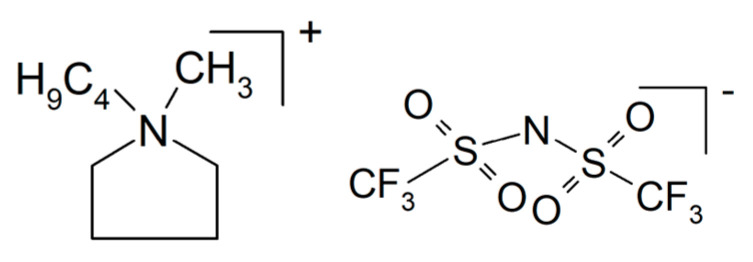
Molecular structures of *N*-butyl-*N*-methylpyrrolidinium bis(trifluoromethyl sulfonyl) imide.

A composite of NaAlCl_4_ with embedded particles of Al_2_O_3_ was prepared by mechanochemical synthesis [149]. The latter provides additional conduction along an oxychloride interface between the two constituents, yielding σ_RT_ > 0.1 × 10^−3^ S/cm. An all-solid-state battery demonstrated electrolyte suitability. An overview on sodium-conducting halides as potential solid electrolyte materials is available [150]. Also, by mechanochemical synthesis, a composite of NaI and Na_2.88_Sb_0.88_W_0.12_S_4_ has been prepared, where the formed solid electrolyte Na_2.88_Sb_0.88_W_0.12_S_4_·0.5NaI showed σ = 3.6.1 × 10^−2^ S/cm and was tested in an all-solid-state battery [151]. For enhanced ion conduction of Na_3_Zr_2_Si_2_PO_12_ (called NACICON instead of NASICON or NASICON-like for unknown reasons), a glass of 35Na_2_O-5Cr_2_O_3_-30TiO_2_-30P_2_O_5_ was infiltrated into the porous ceramic, and use of the product in sodium-ion batteries was proposed [152]. Increased ionic conductivity of Na_3_Zr_2_Si_2_PO_12_ by adding NaF into the precursor mixture during preparation has been reported, where conductivity increased from σ_RT_ = 4.5 × 10^−4^ S/cm to σ_RT_ = 1.7 × 10^−3^ S/cm [153]. A porous pellet of Na_2_ZnSiO_4_ (halloysite) with an ionic liquid *N*-butyl-*N*-methylpyrrolidinium bis(trifluoromethyl sulfonyl) imide (PYR_14_TFSI, see Figure 8) and NaTFSI yielded a solid electrolyte with σ_300°C_ = 1.1 × 10^−3^ S/cm and *t*_+_ = 0.5, suggesting to the authors its use in sodium-ion batteries [154]. A composite of clay and cellulose nanocrystals has been described as a sodium-ion-conducting electrolyte with conductivities around 10^−3^ S/cm, without revealing its preparation details [155]. NaNH_2_ was reacted with B_10_H_14_, yielding solid electrolyte core–shell composites of NaBH_4_ and Na_2_B_12_H_12_ with σ_100°C_ = 1 × 10^−4^ S/cm [156]. A solid-state sodium-ion battery with this composite electrolyte was run for 100 cycles.

##### PVA-Based Materials

To PVA with NaClO_4_ as the salt (presumably not the filler as claimed in the text), Y_2_O_3_ was added as an inorganic filler, affording a solid composite electrolyte with σ_RT_ = 3.25 × 10^−4^ S/cm and *t*_+_ = 0.919 at the optimum composition with 3 wt.% filler (because the total composition of this material was given as 123%, uncertainty remains, and that is also the case regarding other barely comprehensible claims in this report) [106]. High conduction and *t*_+_ were attributed to lower crystallinity caused by the filler and strong interactions between it and the salt anion. For the assembled complete cell, the typical zinc electrode reaction was provided for the negative electrode, and for the positive one, the PbO_2_ and the V_2_O_5_ reactions. The decrease in crystallinity of PVA by adding various amounts of salts like MgSO_4_ or Li_2_SO_4_ has been demonstrated [157]. The effect was more pronounced with the lithium salt. At concentrations beyond around 20 wt.%, crystallization of the added salt starts, and conductivity decreases again.

Addition of sulfuric acid to a mixture (PVA)_0.7_(NaI)_0.3_ yielded a proton-conducting solid electrolyte tested in a sodium/MnO_2_ battery [158]. The addition of acid resulted in σ_RT_ = 10^−3^ S/cm attributed to disruption of the semi-crystalline structure of the mixed solid. Electrical properties of a polymer blend of PVA and methylcellulose with NaI as the electrolyte have been studied [159].

##### PEO-Based Materials

**Figure 9:**
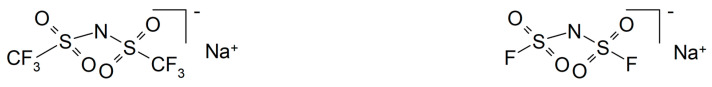
Molecular structures of sodium bis(trifluoromethylsulfonyl) imide NaTFSI and sodium bis(fluorosulfonyl) imide NaFSI.

**Figure 10:**
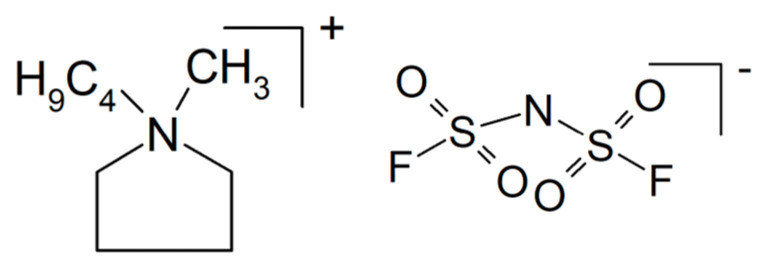
Molecular structures of *N*-butyl-*N*-methylpyrrolidinium bis(fluorosulfonyl) imide PYR_14_FSI.

The notorious tendency of PEO to crystallize (for a study of correlations between crystallinity and ionic conductivity as a function of the fraction of added ceramic filler, see [160]) has been decreased by grafting yielding a hyperbranched polyether, and with NaTFSI (see Figure 9), it showed an increased conductivity σ_RT_ = 5.7 × 10^−4^ S/cm [161]. A cell with a sodium-metal electrode (called a sodium-ion battery) nevertheless kept 92% of its initial capacitance after 300 cycles. A solid composite electrolyte of PEO and NaTFSI with added NASICON-type Na_3.4_Zr_1.8_Mg_0.2_Si_2_PO_12_ with σ_80°C_ = 2.8 × 10^−3^ S/cm was used in a sodium/Na_3_V_2_(PO_4_)_3_ (NVP) battery operated for 80 cycles without significant losses [162]. To a mixture of PEO and NaFSI (see Figure 9) with the mass ratio 20:1 (this is presumably meant with the rather uncommon designation P(EO)_20_NaFSI and later EO:Na = 20:1) the researchers dissolved in acetonitrile various amounts of PYR_14_FSI (see Figure 10), yielding after evaporation of the solvent a solid electrolyte with σ_RT_ = 1.15 × 10^−4^ S/cm (without the ionic liquid σ_RT_ = 2.85 × 10^−6^ S/cm) tested in a sodium-metal/NVP battery at *T* = 60 °C [163]. A capacitance retention 85% after 300 cycles was reported. Conductivity improvement by the added ionic liquid was attributed to increased amorphicity and stronger interactions between NaFSI and the PEO oxygen atoms. With respect to desired sodium-ion transport and electrostatics, presumably interactions between said functionalities and the anion FSI^−^ were meant.

A composite electrolyte was prepared from PEO, NaTFSI, and zeolite A (LTA, presumably its sodium aluminosilicate form) with σ_30°C_ > 1.42 × 10^−4^ S/cm and *t*_+_ = 0.44 and used in a sodium/NVP battery with 88% capacity retention after 100 cycles at *T* = 60 °C [164]. A Cu-metal organic framework on PEO combined with PAN yielded a solid electrolyte with σ_RT_ = 0.103 × 10^−3^ S/cm and *t*_+_ = 0.58 for sodium batteries [165]. A full cell showed a stable performance for 2000 cycles. A Zr-metal organic framework material was combined with PEO, yielding a solid electrolyte for a sodium battery with 97% capacitance retention after 1000 cycles [166]. σ_60°C_ = 6.62 × 10^−4^ S/cm and *t*_+_ = 0.67 were attributed to adsorption of anions on zirconium sites. A composite electrolyte has been prepared from PEO, NaCF_3_SO_3_, and as a filler MOF MIL-53(Al) and has been used in a sodium/sulfur battery [167]. At 3.24 wt.% of the filler, *t*_+_ = 0.4 and σ_50°C_ = 1.5 × 10^−5^ S/cm were recorded, and without filler, the value decreased by almost an order of magnitude. Operated at *T* = 60 °C, the battery kept 75% of the initial capacitance after 50 cycles. Promising results for lithium/sulfur batteries obtained with a cubic-garnet Li_7_La_3_Zr_2_O_12_ applied in a bilayer arrangement to ameliorate electrolyte/lithium incompatibilities may indicate a direction of further development [168].

The problem of agglomeration of one (mostly the inorganic) constituent in a polymer/inorganic filler composite electrolyte has been addressed in detail [169] and experimentally verified for some composites [170], and similar observations with composites of polymers with graphene, graphene oxide, etc., are discussed elsewhere [171]. Surface modification of the inorganic filler Na_3_Zr_2_Si_2_PO_12_ with polydopamine improved the wettability, with the polymer component PEO enabling an all-solid-state sodium-metal battery running stably for 1350 cycles at *T* = 60 °C. According to results of modeling the surface modification of the inorganic filler, this also influenced sodium ion transport, helping to suppress dendrite formation. An optimized Na_3.3_Zr_1.7_La_0.3_Si_2_PO_12_ with σ_RT_ = 3.4 × 10^−3^ has been used in a sodium-metal battery [172]. For improved electrode/electrolyte interaction at the positive electrode, a small amount of organic liquid electrolyte or ionic liquid was soaked into the porous electrode body. Following the initial considerations provided above, no reason to call this a composite electrolyte is evident.

**Figure 11:**
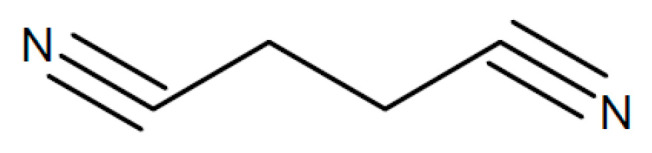
Molecular structures of succinonitrile (SN).

**Figure 12:**
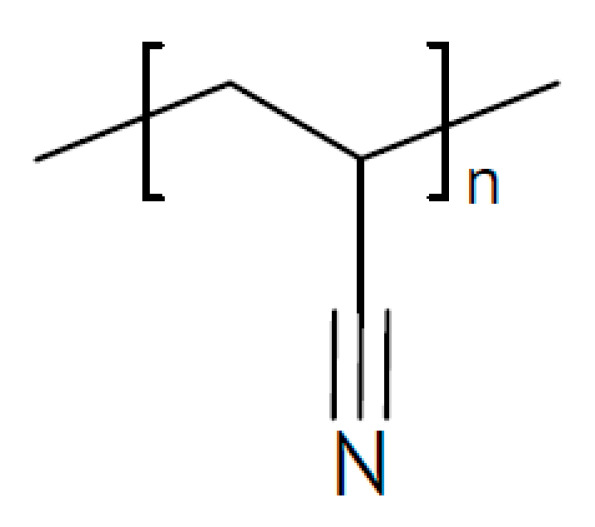
Molecular structures of polyacrylonitrile.

A laminated two-layer electrolyte has been proposed for sodium-ion batteries [173]. Facing the negative sodium-metal electrode a PEO film with added succinonitrile (see Figure 11) for better sodium-ion conduction is placed, and on the other side, there is a polyacrylonitrile (PAN) film (see Figure 12) with added NASICON-type Na_3_Zr_2_Si_2_PO_12_ for the same purpose. A value σ_RT_ = 1.36 × 10^−4^ S/cm has been reported.

An asymmetric composite electrolyte with a harder (or tougher) side facing the sodium-metal electrode (for better dendrite suppression) and a softer side for the positive electrode, enabling formation of a better interface, has been proposed [174]. The tougher side was prepared from PEO, NaTFSI, and a metal–organic framework ZIF-67, and upon this, more PEO/NaTFSI was deposited. Overall conductivity was σ_RT_ = 5.23 × 10^−4^ S/cm, and a complete cell showed 0.12% capacitance loss per cycle for 300 cycles. Mixtures of PEO and NaTFSI have been studied, and with growing fraction of the salt, the mix becomes more gum-like, suggesting plasticizer-like properties of the salt [175]. Somewhat surprisingly—when considering the effects of this filler in other composites—5 wt.% of nanosized SiO_2_ did not change the ionic conductivity. The interfacial resistance with the sodium electrode slightly increased, where *t*_+_ increased from *t*_+_ = 0.39 to *t*_+_ = 0.51. A membrane made from a mixture of a PEO-like polymer and Al_2_O_3_ (2:1 by weight) soaked in a solution of 1 M NaClO_4_ in a carbonate solvent mixture yielded a composite electrolyte tested in a sodium/Na_2_MnFe(CN)_6_ battery [62]. The filler blocked dendrite formation, and the cell showed no capacitance loss after 400 cycles. Suitability of a composite electrolyte prepared from PEO and NaPF_6_ with TiO_2_ (4 wt.%) as a filler for a solid-state sodium battery Na/NaTi_2_(PO_4_)_3_ has been studied [176]. Without filler, σ_RT_ ~ 0.013 × 10^−3^ S/cm, and at *T* = 80 °C, 0.2 × 10^−3^ S/cm was measured (for comparison, see [177]); upon filler addition, the latter value increased to 0.44 × 10^−3^ S/cm with *t*_+_ = 0.68, and at T = 80 °C, the battery showed a stable capacitance during 110 cycles. A solid composite electrolyte of PEO with NaClO_4_ as a salt and 5 wt.% TiO_2_ as a filler showed an increase in the conductivity from σ_RT_ = 1.35 × 10^−4^ S/cm to σ_RT_ = 2.62 × 10^−4^ S/cm and good compatibility with an electrode of Na_2/3_Co_2/3_Mn_1/3_O_2_ [178].

Addition of 50% succinonitrile (SN, Figure 11) to a solid electrolyte of PEO and NaTFSI increased the ionic conductivity to σ_RT_ ~ 1.1 × 10^−4^ S/cm, about 45 times the value without SN [179]. SN has been called a “non-ionic plastic crystal” apparently because it is an organic crystalline solid at room temperature, with specific properties found in more details elsewhere [180,181].

A composite of PEO, NaTFSI, and Na_6_[Re_4_As_2_S_2_(CN)_12_] with a cubane-like anion showing σ_RT_ = 1.05 × 10^−5^ S/cm was tested successfully in a sodium/NaVPO_4_ cell [182]. A composite of PEO with partially hydrolyzed NaBH_4_ has been examined as a solid electrolyte for a sodium-ion battery [183]. σ_45°C_ = 11.6 × 10^−3^ S/cm and *t*_+_ = 0.54 were found. Functionalized carbon carboxylate composited with sodium bis(oxalate)-borate yielding an electrolyte for sodium-ion batteries has been prepared [184]. A porous disk of alumina was soaked with a slurry of PEO, SiO_2_, and NaClO_4_ (for its preparation, see [185]), yielding a solid composite electrolyte with σ_RT_ = 1.6 × 10^−4^ S/cm for a sodium-metal battery (although according to a displayed figure, the electrolyte is permeable for lithium ions!) [186]. A ceramic/polymer composite has been proposed for use in a sodium-ion battery at *T* = 80 °C [187]. NASICON-type powders of Na_3_Zr_2_Si_2_PO_12_/Na_3.4_Zr_1.8_Mg_0.2_Si_2_PO_12_ as the ceramic constituent were combined with PEO (see Figure 4) and NaTFSI, where the obtained film was sandwiched between the negative sodium ion and the positive Na_3_V_2_(PO_4_)_3_ electrode. Sodium-ion conductivity of 2.4 mS·cm^−1^ was reported. PEO with NaTFSI was composited with Na_3_Zr_2_Si_2_PO_12_, yielding a composite electrolyte with σ_RT_ = 1.4 × 10^−4^ S/cm at the optimum composition [188]. A solid electrolyte based on Na_3_Zr_2_Si_2_PO_12_ and PEO with NaClO_4_ and some PEG “for better film formation” and σ_55°C_ > 10^−4^ S/cm has been described [189]. A cell with a sodium-metal electrode kept 98% of the initial capacitance after 100 cycles. The same composite electrolyte was used in a sodium-ion battery of symmetric design with two Na_3_V_2_(PO_4_)_3_, showing 86.4% of its initial capacitance after 650 cycles [190]. When using a positive Prussian blue electrode instead, 0.005% capacity loss per cycle during 9000 cycles was observed. To PEO with NaClO_4_ as a salt, Na_3_Zr_2_Si_2_PO_12_ has been added, affording a solid composite electrolyte with increased conductivity σ_30°C_ = 2.1 × 10^−5^ S/cm and enhanced dendrite suppression [191]. It was used in a sodium-metal-Prussian Blue-type positive electrode battery providing 0.05% capacitance loss per cycle during 300 cycles. To a solid electrolyte of PEO with sodium percarbonate, Na_3_Zr_2_Si_2_PO_12_ was added, yielding a conductivity σ_RT_ = 2.6 × 10^−4^ S/cm (the unit SCm^−1^ remains a mystery) [192]. High conductivity was attributed to lower crystallinity, and sodium dendrite formation was suppressed, yielding 73% capacitance retention after 100 cycles. A polyether-based composite electrolyte with NaTFSA as a salt and Na_3_Zr_2_Si_2_PO_12_ as an inorganic filler showed increasing conductivity with a decreasing filler content, e.g., σ_RT_ = 1.03 × 10^−5^ S/cm (*t*_+_ = 0.04) at 30 wt.% filler [193]. This behavior was attributed to improved segmental mobility of the polyether at a lower filler content, promoting dissociation of the added salt. A PEO-based solid electrolyte with added NASICON-type Na_3_Zr_2_Si_2_PO_12_ doped with Sc and Ge and enriched ceramic content near the electrolyte surface has been used in a sodium-ion battery [143]. Overall conductivity of the electrolyte was σ_30°C_ = 4 × 10^−5^ S/cm. A complete cell with a negative electrode Sn_4_P_3_CNT/C and a NVP positive one provided 100 cycles with 88% capacitance retention. A further increase in sodium-ion conductivity as well as transference number *t*_+_ were obtained by doping NASICON-type ceramic Na_3_Zr_2_Si_2_PO_12_ with Mg^2+^ and Sc^3+^; similar to the preceding example, the ceramic was mixed with NaTFSI and PEO, yielding a composite electrolyte with *t*_+_ ≈ 0.998 and σ_RT_ = 7.96 × 10^−5^ S/cm were reported with an optimized composition of the ceramic [194]. A full sodium ion cell was operated at *T* = 60 °C stably for 80 cycles. Using as a salt NaClO_4_ instead, a cell with a sodium-metal electrode retaining 97% of its initial capacitance after 100 cycles was reported [195]. PEO with NaClO_4_ added as a salt was infiltrated into a skeleton of electro-spun Na_3_Zr_2_Si_2_PO_12_, yielding σ_RT_ = 4.43 × 10^−4^ S/cm and *t*_+_ = 0.61 [196]. A full cell with a sodium electrode kept 83% of its initial capacitance after 1500 cycles. A composite of this ceramic with polyethylene glycol diacrylate and succinonitrile as a plasticizer has been suggested as an electrolyte for sodium batteries [197]. The ceramic enhanced ionic conductivity (σ_RT_ = 4.5 × 10^−4^ S/cm) and suppressed dendrite formation, contributing to 87% capacitance retention after 100 cycles.

A glass fiber mat coated with polyethylene glycol diacrylate was soaked with PEG in the presence of NaClO_4_, yielding a composite electrolyte (σ_RT_ = 0.8 × 10^−4^ S/cm), and tested in a Na/NVP battery, showing 99% capacity retention after 1100 cycles [198].

A solid electrolyte of PEO with NaTFSI as a salt and hydroxyapatite added as a filler, yielding about σ_70°C_ ~ 10^−4^ S/cm and *t*_+_ = 0.38, was tested in a sodium/Na_4_CrFe(PO_4_)_3_ battery [199]. A composite of PEO with NaClO_4_ with BaTiO_3_ filler for a sodium-ion battery has been reported [114]. At 5 wt.% filler, σ_RT_ ~ 1 × 10^−5^ S/cm was reported, and reduced crystallinity was noted as the cause of increased conductance. Addition of electrospun fibers of MgAl_2_O_4_ to PEO with NaClO_4_ yielded a composite electrolyte with increased ionic conductivity (σ_55°C_ = 1.89 × 10^−4^ S/cm with *t*_+_ = 0.55) and higher mechanical and thermal stability [200]. The increased conductivity is attributed to lower crystallinity and stronger interaction between the filler and the anions of the salt.

To PEO with NaTFSI as salt, Ga-doped Na_2_Zn_2_TeO_6_ filler at various concentrations was added, yielding a solid composite electrolyte with σ_30°C_ = 4 × 10^−5^ S/cm at 50 wt.% filler from σ_30°C_ = 1 × 10^−6^ S/cm without filler, with the increase ascribed to the high ionic conductivity of the filler itself and suppressed crystallization [201]. A sodium/NVP battery was assembled; at *T* = 80 °C, cells with both micro- and nanosized NVP lost about 10% of the initial capacity during 100 cycles.

A PEO-based (not cellulose-based as claimed in the title) polymer electrolyte with NaClO_4_ as a salt blended with sodium carboxymethyl cellulose (CMC) has been prepared, characterized, and tested in Na/TiO_2_ and Na/NaFePO_4_ batteries [202]. The composite showed σ_RT_ < 10^−6^ S/cm and *t*_+,60°C_ = 0.15, and batteries were run for 12 and 20 cycles, respectively.

**Figure 13:**
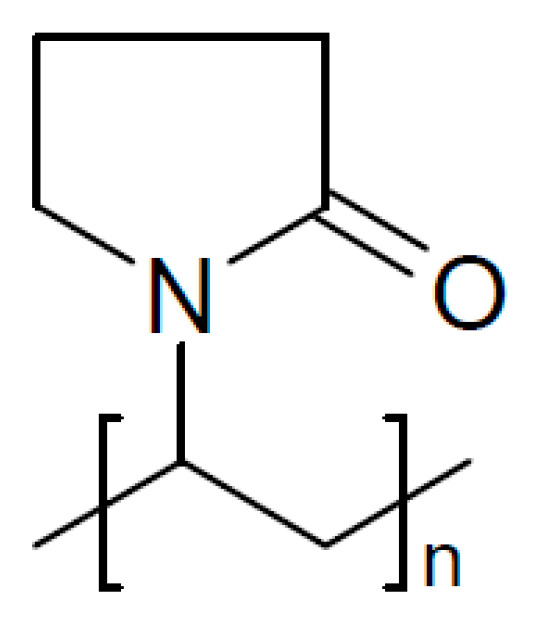
Molecular structures of polyvinyl pyrrolidone.

A polymer blend of PEO and polyvinyl pyrrolidone (PVP, Figure 13) with NaIO_4_ as a salt composited with TiO_2_ nanoparticles has been prepared for a sodium [203]. At 10 wt.% salt and 3 wt.% filler, σ_RT_ = 9.82 × 10^−6^ S/cm was found, and as a reason, the increase in the dielectric constant of the material causing an increase in the concentration of mobile ions and their mobility was suggested.

Ionic conductivity of a PEO/PVP blend with added NaF has been studied, where a maximum conductivity σ_30°C_ = 1.19 × 10^−7^ S/cm was reported [204]. A polymer blend of PEO and PEMA with NaIO_4_ with ZnO as a filler and PEG as plasticizer has been prepared and studied [205]. Growing filler content resulted in higher conductivity.

**Figure 14:**
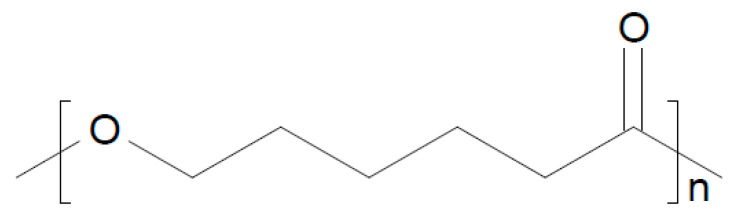
Molecular structures of polycaprolactone.

To a blend of PEO/polycaprolactone (see Figure 14), graphitic C_3_N_4_ and Na-β-Al_2_O_3_ have been added, yielding a composite electrolyte tested at *T* = 50 °C in a Na/NVP battery, showing a stable capacity for 55 cycles [206].

**Figure 15:**
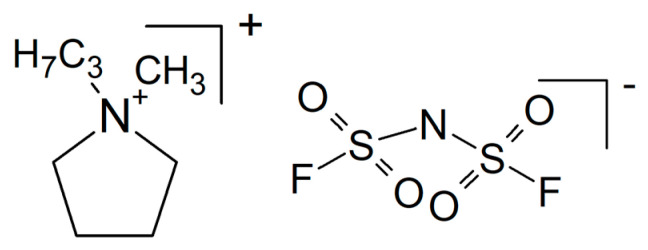
Molecular structures of *N*-propyl-*N*-methylpyrrolidinium bis(fluorosulfonyl) imide.

Although inhibition of crystallization by adding a plasticizer seems to be a popular and well-established approach to soften hard and/or brittle plastics, the title of a report highlighting plasticizing as something special suggests otherwise [207]. Adding *N*-propyl-*N*-methylpyrrolidinium bis(fluorosulfonyl)imide (PYR_13_FSI, see Figure 15) as a plasticizer to PEO with NaClO_4_ as an electrolyte yielded a composite electrolyte (with this term used generously in a somewhat wider sense, see above) with σ_RT_ = 6.8 × 10^−5^ S/cm and *t*_+_ = 0.44 at 40 wt.% of the ionic liquid. A Na/NVP battery kept 86% of the initial capacity after 70 cycles.

##### PVDF-Based Materials

A PVDF-based composite electrolyte with NaTFSI as a salt and an inorganic filler of *β*″-Al_2_O_3_ has been prepared, characterized, and tested in Na/CF_x_ and Na/NaNi_1/3_Mn_1/3_Fe_1/3_O_2_ batteries [208]. σ_RT_ = 4.55 × 10^−4^ S/cm, and the six-fold increase in comparison to the conductivity of the solid electrolyte without filler was attributed to a “destruction of the ordered arrangement of PVDF chains”, i.e., reduced crystallinity. Lower Coulomb efficiency of the solid-state cell in comparison to a conventional cell with liquid electrolyte solution was attributed to a poor electrolyte/electrode interface in the solid-state cell, as already addressed above. Capacity retention was 87% after 50 cycles for the Na/NaNi_1/3_Mn_1/3_Fe_1/3_O_2_ battery. Na/CF_x_ cells with carbon fluorides of different degrees of fluoridation performed poorly.

To PVDF with succinonitrile as a plasticizer and NaTFSI as a salt, Na_3.4_Zr_1.8_Ni_0.2_Si_2_PO_12_ was added for improved sodium-ion conduction (σ_RT_ = 1.02 × 10^−3^ S/cm), yielding a solid electrolyte for a sodium-metal battery with 90% capacitance retention after 700 cycles [209]. A solid electrolyte of PVDF with Na_3_Zr_2_Si_2_PO_12_ and NaClO_4_ as salt (σ_RT_ = 1.1×10^−4^ S/cm, for plain PVDF σ_RT_ = 3.1 × 10^−5^ S/cm) was used as a composite electrolyte in a sodium metal/Na_0.67_MnO_2_ battery [210]. Wetting the porous positive electrode with a very small amount of a liquid organic electrolyte solution helped in establishing a good electrode/electrolyte interface. At an optimized content of liquid a capacitance retention of 100% after 100 cycles was observed. A solid electrolyte of PVDF with NaCF_3_SO_3_ as salt and SiO_2_ as a filler yielded a solid composite electrolyte with σ_RT_ = 0.06 × 10^−3^ S/cm [211]. The complete Na/NVP battery kept 70% of its initial capacitance after 100 cycles, for better contact between electrolyte and positive electrode some liquid electrolyte solution was added to the latter.

Cyclic stability was attributed to prevented dendrite formation at the sodium electrode and inhibited manganese dissolution at the positive electrode. Electrospun fibers of PVDF/NaFSI with a shell of Na_3_Zr_2_Si_2_PO_12_ were made into a separator/composite electrolyte with σ_60°C_ = 6.6 × 10^−4^ S/cm, enabling a sodium-metal battery showing relatively poor specific capacity, attributed to the poor electrode/electrolyte contact [212]. Into a solid electrolyte of PVDF with NaClO_4_ as a salt Na_3_Zr_2_Si_2_PO_12_ was added, yielding a composite electrolyte with σ_RT_ = 1.069 × 10^−4^ S/cm tested in a sodium-ion battery keeping 95% of its initial capacitance after 290 cycles [213]. Into a solid electrolyte of PVDF with NaPF_6_ sodium β”, alumina was added [214]. The modified composite electrolyte had σ_RT_ = 0.19 × 10^−3^ S/cm and *t*_+_ = 0.91, and a complete sodium/carbon nanofiber (CNF) cell kept 95% of its initial capacitance after 100 cycles. A composite of this ceramic with PVDF-HFP with “Salt-Ionic liquid” (this apparently means an ionic liquid EMIMTFSI as a plasticizer and NaTFSI as a salt) has been prepared, yielding σ_RT_ = 1 × 10^−3^ S/cm and *t*_+_ = 0.57 [215].

A composite electrolyte was obtained by coating both sides of a glass fiber mat first with PVDF-HFP and then with polydopamine and by finally soaking the material in a 1 M solution of NaClO_4_ [216]. σ_RT_ = 4.6 × 10^−3^ S/cm was reported, and with polydopamine coating, the value increased to σ_RT_ = 5.4 × 10^−3^ S/cm; a sodium/Na_2_MnFe(CN)_6_ battery showed 89% capacity retention after 100 cycles.

Into a porous skeleton of PVDF-HFP and Na_3_Zr_2_Si_2_PO_12_, an interpenetrating network of poly(ether-acrylate), PEO, and NaPF_6_ was embedded, providing a composite electrolyte with σ_60°C_ = 1.32 × 10^−4^ S/cm and *t*_+_ = 0.63 at *T* = 60 °C [217]. Operated at this temperature, sodium-metal cells and various positive electrode materials showed stable capacities over 100 cycles. The multilayer structure of the electrolyte frequently claimed in the report is nowhere evident.

**Figure 16:**
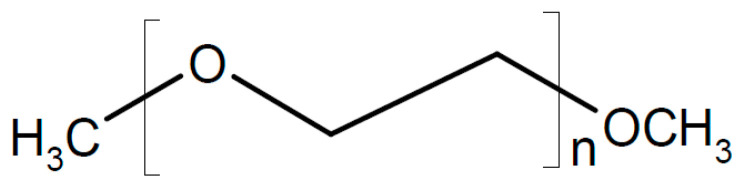
Molecular structures of tetraethylene glycol dimethylether (TEGDME).

A solid electrolyte Na_3_Zr_2_Si_2_PO_12_ with added BaTiO_3_ (in the abstract, it is called reinforcement, but in the report, this claim does not show up again) better prevents dendrite formation, enabling a full cell to keep 84.4% of its initial capacitance after 400 cycles [218]. A composite sheet of Na_3_Zr_2_Si_2_PO_12_ and PVDF-HFP soaked in an electrolyte solution of sodium triflate and TEGDME (presumably tetraethylene glycol dimethyl ether, see Figure 16) showed σ_0°C_ = 1.2 × 10^−4^ S/cm, σ_RT_ = 3.6 × 10^−4^ S/cm, and *t*_+_ = 0.92 [32].

In a complete battery, only a slight capacitance loss was observed over 200 cycles.

A composite of PVDF-HFP and Na_3_Zr_2_Si_2_PO_12_ soaked in a solution of 1 M NaPF_6_ in a mixed carbonate solvent was used as an electrolyte (σ_RT_ = 7.94 × 10^−4^ S/cm) in a Na/Sn-C battery [219]. The high conductivity was attributed to the liquid electrolyte solution trapped inside the porous composite. After initial major capacity losses (about 50%), the cell capacity stayed constant for the following 80 cycles.

**Figure 17:**
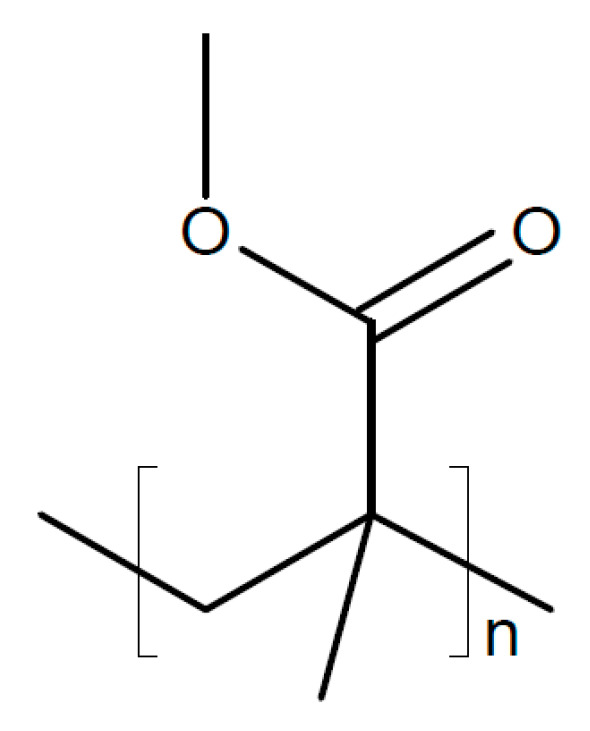
Molecular structures of poly(methyl methacrylate) (PMMA).

PVDF-HFP coated on both sides of a disk of Na_2.5_Zr_1.95_Ce_0.05_Si_2.2_P_0.8_O_11.3_F_0.7_ (σ_RT_ = 1.7 × 10^−3^ S/cm) was used as a solid electrolyte in a sodium-metal/Na_0.67_Mn_0.47_Ni_0.33_Ti_0.2_O_2_ battery, showing a stable performance over 300 cycles [220]. A similar ceramic Na_3.2_Zr_1.9_Ca_0.1_Si_2_PO_12_ combined with PVDF-HFP showing σ_RT_ = 1.32 × 10^−4^ S/cm has been suggested as solid electrolyte for a sodium/CO_2_ battery [221]. A solid composite electrolyte was prepared from PVDF-HFP with NaClO_4_ and Na_3.2_Zr_1.9_Mg_0.1_Si_2_PO_12_ added as a filler for use in a sodium/CO_2_ battery [222]. Partial substitution of Zr by Mg resulted in σ_RT_ = 1.16 × 10^−3^ S/cm. A polymer composite membrane of PVDF-HFP and Na_3_Zr_2_Si_2_PO_12_ filled with poly(methyl methacrylate) (PMMA, see Figure 17) was used as a solid electrolyte with σ_RT_ = 2.78 × 10^−3^ S/cm and *t*_+_ ~ 0.63, which enabled a stable cycling performance of a sodium-ion battery for 600 cycles [223].

A composite of PVDF-HFP as the matrix and a sodium-rich anti-perovskite/perovskite material has been suggested as electrolyte for sodium batteries [224]. σ_RT_ = 1.11 × 10^−4^ S/cm and stability over 500 cycles were reported. To improve the low-temperature ionic conductivity of PVDF-HFP (not PVDF as suggested in the title of the report) with NaClO_4_ as the salt, graphitic C_3_N_4_ has been added [225]. This addition increased conductivity by enhancing salt dissociation and reducing crystallinity of the copolymer. σ_RT_ = 5.171 × 10^−5^ S/cm without g-C_3_N_4_ increased to σ_RT_ = 1.67 × 10^−4^ S/cm, and *t*_+_ improved from 0.61 to 0.78. In a sodium-metal battery, 98% of the initial capacitance was retained after 200 cycles; with the addition, this decreased to 69%. A polymer blend of PVDF-HFP and PMMA was dissolved and β-alumina nanopowder was added, possibly (the report leaves this detail open) the blend was soaked in a 1 M NaClO_4_ in a blend of carbonate solvents [226]. Solution uptake was highest at 4 wt.% filler content, conductivity was highest at this composition with σ_RT_ = 3.39 × 10^−3^ S/cm attributed to lower crystallinity, *t*_+_ = 0.58 was attributed to a weaker interaction between fluoride atoms in the polymer and sodium ions caused by the added filler. In a test with this composite electrolyte a sodium/Na_3_V_2_(PO_4_)_3_ battery kept 85% of its initial capacitance after 300 cycles.

To a polymer blend of PVDF-HFP and PMMA with NaCF_3_SO_3_ dissolved in a carbonate solvent mixture presumably also acting as a plasticizer and as a salt, Al_2_O_3_ was added as a filler, providing a gel electrolyte with σ_RT_ ~ 1.5 × 10^−3^ S/cm and *t*_+_ ~ 0.29. 0.33 (not *T* as reported) at a 6 wt.% optimum filler content [227]. Enhanced conductivity caused by addition of PMMA was attributed to the amorphous nature of this polymer, whereas the added filler increased salt dissociation. PVDF-HFP combined with β-Al_2_O_3_ powder surface modified with silane for better compatibility with the polymer matrix yielded a solid electrolyte after soaking with an electrolyte solution of NaPF_6_ in a mixed carbonate solvent for a sodium-metal battery with σ_20°C_ = 1.37 × 10^−3^ S/cm and *t*_+_ = 0.424, enabling 92% capacitance retention after 1000 cycles [228]. A copper-based MOF HKUST-1 [229] was used as inorganic filler for PVDF-HFP (confusingly named PH), and the obtained membrane soaked in a 1 M NaClO_4_ solution in ethylene carbonate with 5% fluoroethylene carbonate yielded a composite electrolyte with σ_30°C_ = 3.2 × 10^−4^ S/cm and *t*_+_ = 0.64 [230]. When tested in a sodium/Na_3_V_2_O_2_(PO_4_)_2_ battery, 75% of the initial capacitance was kept after 400 cycles. Inhibition of dendrite formation was attributed to the inorganic filler; in addition, the filler enhanced liquid electrolyte solution absorption and amorphicity.

A ternary polymer blend of PVDF-HFP, PEO, and PMMA has been combined with 10 wt.% silica fillers and soaked in an electrolyte solution of NaPF_6_ in propylene carbonate, yielding a composite electrolyte subsequently tested in a sodium/Na_3_V_2_(PO_4_)_2_F_3_ [231]. σ_RT_ = 0.88 × 10^−3^ S/cm and *t*_+_ = 0.86 were found, or without filler, only σ_RT_ = 0.63 × 10^−3^ S/cm and *t*_+_ = 0.74. The cell kept 93% of its initial capacitance after 100 cycles.

Using Sb_2_O_3_ instead of Al_2_O_3_ as a filler for a polymer mixture of PVDF-HFP and PVP yielded a highly flexible composite membrane, which was soaked with an electrolyte solution of 1 M NaClO_4_ in a carbonate solvent mixture [232]. The result, called a separator, had a “large electrolyte window”—whatever that means, it blocked dendrite formation. The same approach has been reported with SiO_2_ as a filler material [233]. Here, 6 wt.% filler was found as the optimum fraction, yielding an electrolyte with σ_RT_ = 0.71 × 10^−3^ S/cm and stable performance in a symmetric Na//Na cell for 200 cycles. Into a polymer blend PVDF-HFP and PEO with NaClO_4_ as salt and EMIMFSI as plasticizer, microtubular Na_3_Zr_2_Si_2_PO_12_ was added, yielding a solid electrolyte with σ_RT_ = 6.93 × 10^−4^ S/cm and *t*_+_ = 0.882 for a sodium-metal battery [234].

Nanoparticles of a zeolitic imidazolate framework ZIF-67 impregnated into a fibrous membrane of PVDF-HFP and PAN yielded a sodium-ion-conducting membrane with σ_RT_ = 1.42 × 10^−3^ S/cm and *t*_+_ = 0.58 [235]. The interface established between this membrane and the sodium-metal electrode prevented sodium dendrite formation during cycling. A complete cell showed 0.052% capacitance decline per cycle during 100 cycles.

##### Other Polymer-Based Materials

**Figure 18:**
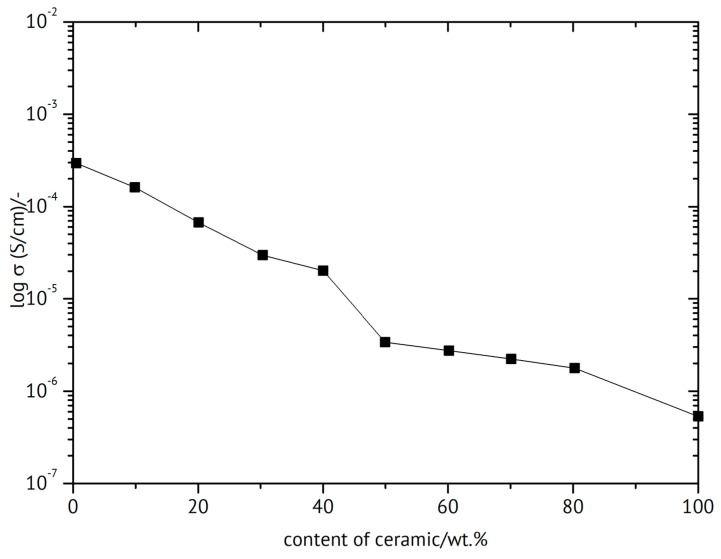
Ionic conductivity of a ceramic/polymer composite as a function of composition at room temperature, based on data in [237].

An overview of polymer electrolytes focused on sodium batteries is available [236]. An epoxy-reinforced ceramic sheet of NASICON type of Na_3_Zr_2_Si_2_PO_12_ has been suggested for a solid-state sodium-ion battery [237]. The beneficial effect of the added polymer beyond improving mechanical strength is illustrated in Figure 18, showing the ionic conductivity as a function of the polymer/ceramic composition. The porous sintered ceramic pellet was filled with the polymer. Sintering of the inorganic components before combining the constituents was found to be highly advantageous in terms of conductivity of the product.

**Figure 19:**
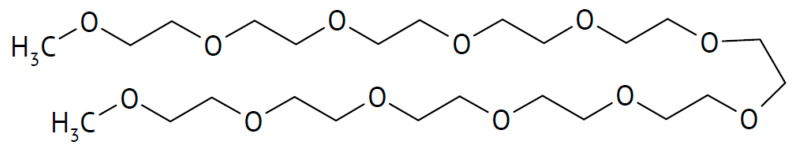
Molecular structures of polyethylene glycol dimethylether.

A highly sodium-ion-conducting SiO_2_/polymer hybrid has been reported [238]. For an increased value of *t*_+_, anions were immobilized by grafting anions of 2-[(trifluoromethane-sulfonylimido)-N-4-sulfonylphenyl]ethyl onto SiO_2_ particles, and this hybrid material was dispersed in a polymer matrix of polyethylene oxide (PEO, see Figure 4) or polyethylene glycol dimethylether (PEGDME, see Figure 19), yielding a composite electrolyte.

Conductivities σ_RT_ > 10^−5^ S/cm were found. A matrix of electrospun SiO_2_ fibers was filled with a copolymer of 2-(methacryloyloxy) ethyl acetoacetate and *N*,*N*′-methylenebisacrylamide, yielding a composite electrolyte with σ_−30°C_ = 0.153 × 10^−3^ S/cm [239]. A final coating with AlF_3_ suppressed sodium dendrite formation. Capabilities of this electrolyte were demonstrated by 5500 h steady sodium plating/stripping. How a pouch half-cell (elsewhere in the text, full cell) was powered by several LEDs was not explained; a cell kept 94% of its initial capacitance after 475 cycles. With Al_2_O_3_ as a filler, an acrylate-based polyester polymer yielded an electrolyte for a sodium-metal battery with σ_RT_ = 5.59 × 10^−3^ S/cm [240]. A composite of polysulfone-sodium sulfonate and poly(polyethylene glycol methacrylate) with nanosized hexagonal boron nitride for sodium-ion batteries has been reported [241]. σ_100°C_ = 5.5 × 10^−5^ S/cm was noted.

A copolymer of acrylonitrile and polyethylene glycol methacrylate with NaClO_4_ as the salt and hexagonal boron nitride as the filler has been developed [242]. An optimum composition of σ_?_ = 3.6 × 10^−4^ S/cm at an unknown temperature (possibly, according to a displayed figure, *T* = 100 °C) was reported, and the proposed use in a sodium-ion battery was not described. A composite electrolyte of poly(ethylene glycol) diacrylate (see below) reinforced with glass fibers and σ_?_ = 1.38 × 10^−3^ S/cm/*t*_+_ = 0.79 has been tested in a sodium-metal battery, yielding 91% capacitance retention after 1000 cycles [243]. Felts of electrospun ceramic fibers surface-modified with acyl amino groups for enhanced interfacial sodium ion transport were filled with a deep eutectic electrolyte for long-range ion transport [244]. A value of σ_26°C_ = 3.29 × 10^−4^ S/cm and 98% capacity retention after 1000 cycles were reported. Cross-linked β-alumina nanowires provided the scaffold for a PVDF-HFP-based gel polymer electrolyte in a sodium-metal battery [245]. The mechanical rigidity of the electrolyte supported smooth sodium deposition, resulting in 95% capacitance retention after 1000 cycles.

A gel polymer composite electrolyte prepared from cellulose triacetate and a polyionic liquid has enabled a sodium-metal cell to run for more than 800 cycles [246]. How the electrolyte powered the cell was not explained. A PEO-based gel polymer electrolyte with 1-butyl-1-methylpyrrolidinium bis(trifluoromethylsulfonyl) imide as the gelling agent and NaTFSI as the salt was modified with surface-modified sepiolite [247]. Conductivities’ depended on ionic liquid content, with large fractions of the liquid σ_RT_ = 0.95 × 10^−3^ S/cm found, which, with lower fractions this value, decreased to 0.39 × 10^−3^ S/cm. The influence of sepiolite on ionic conductivity was rather moderate.

A NASICON-type sodium-ion conductor of unspecified composition with a dense core and porous outer layers has been prepared [248]. The porous layer facing the sodium electrode was filled with PEO, and the one facing the positive Na_3_V_2_(PO_4_)_2_F_3_ with PAN for better compatibility with the respective electrodes. σ_30°C_ = 4.3 × 10^−4^ S/cm was attributed to long-range ionic pathways, and lower interfacial resistance to the better compatibility between electrodes and the modified inorganic solid. A full cell provided 81% capacity retention after 460 cycles.

A sodium ion-conducting solid electrolyte based on PEG and NaBr with 5 nm silica nanoparticles has been developed [249]. At 5 wt.%, added silica conductivity enhancement was largest at σ_RT_ = 8 × 10^−5^ S/cm. Adsorption of anions (i.e., Br^−^) on the silica was upheld as the main reason of conductivity increase.

A solid electrolyte of a copolymer poly(ethylene glycol)-co-ureidopyrimidinone with NaTFSI as the salt was modified by adding hollow mesoporous SiO_2_, yielding a solid composite electrolyte with σ_RT_ = 2.37 × 10^−5^ S/cm and *t*_+_ = 0.17 [250]. A sodium/NVP battery assembled with this electrolyte kept 77% of its initial capacity after 200 cycles at *T* = 60 °C, and increased conductivity was ascribed to fast ion pathways along the surfaces of the silica spheres.

Poly(diallyldimethylammonium) bis(fluorosulfonyl)imide with NaFSI (see Figure 9) as the salt dissolved in PYR_14_TFSI (see Figure 8) soaked into a glass fiber mat yielded a composite electrolyte with σ_70°C_ = 2.1 × 10^−3^ S/cm, and when tested in a Na/NVP battery, 75% of its initial capacity was retained after 2000 cycles [251].

Addition of an antioxidant 4-trifluoromethylphenylboronic acid to a cross-linked gel polymer electrolyte helped to avoid electrolyte decomposition at the positive electrode of a high-voltage sodium-ion battery [252].

A composite of Na_3_Zr_2_Si_2_PO_12_ with cellulose acetate obtained by a simple solution-casting method with σ_RT_ = 1.73 × 10^−3^ S/cm was tested in a solid-state sodium battery, enabling 80% capacity retention after 800 cycles [253].

**Figure 20:**
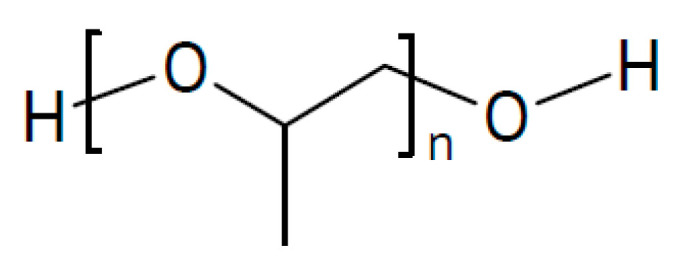
Molecular structures of poly(propylene glycol).

For a sodium-metal battery, a solid electrolyte of porous Na_3_SbS_4_ filled with a polymer PPP of PEG and PPG (the meaning of the acronyms is nowhere revealed; evidence suggests that PPP may be a mixture of poly(ethylene glycol) (Figure 5) and poly(propylene glycol) (Figure 20) with NaTFSI as salt has been proposed [254]. The performance of the sodium-metal electrode was significantly improved, enabling operation for 550 cycles.

Na_3_SbS_4_ is a good sodium-ion conductor but unfortunately chemically incompatible with sodium metal [255]. Nanoparticles of this material coated with an ionic liquid BMPTFSI (1-butyl-1-methylpyrrolidinium bis(trifluoromethylsulfonyl) imide, see the common acronym PYR_14_TFSI Figure 8) were soaked into a porous PVDF membrane, subsequently serving as separator and solid electrolyte, yielding a conductivity σ_RT_ = 3.18 × 10^−3^ S/cm and enabling a good cell performance over 400 cycles. In another attempt to utilize this sodium-ion conductor, the sulfide is deposited onto a glass fiber network, and the product is soaked with an ionic liquid [256]. Good cycling behavior both of the sodium-metal electrode and the positive FeS_2_ electrode in contact with this electrolyte were reported. Another approach to utilize the attractive conductivity of this sulfide by combining it with sodium carboxymethyl cellulose (7.5 wt.% and 92.5 wt.% sulfide) has been reported [257]. Because the thickness of the electrolyte could by decreased to 1/5 in comparison to the sulfide alone, effective sodium-ion conduction grew roughly 5-fold. A sodium/Te-C cell showed about 40% capacitance loss after 100 cycles. A hybrid composite electrolyte prepared from Na_3_SbS_4_ and cross-linked pentaerythritol tetra acrylate with σ_RT_ = 0.047 × 10^−3^ S/cm has been tested in a Na_3_SbS_4_/S cell running for 90 cycles [258].

Air stability of ion-conducting sulfides has been reviewed [259]. In a further attempt, Na_3_SbS_3_Se nanoparticles were embedded in a PVDF-HFP polymer matrix with NaPF_6_ as a salt, yielding a composite electrolyte with σ_RT_ = 1.31 × 10^−4^ S/cm at an optimum sulfide content 10 wt.% [260]. A Na/TiS_2_ battery kept 56% of its initial capacitance after 300 cycles. A core/shell composite has been proposed for use in solid-state sodium batteries [261]. A core of Na_3_PS_4_ is coated with a shell of Na_2.25_Y_0.25_Zr_0.75_Cl_6_, yielding σ_?_ = 0.44 × 10^−3^ S/cm at an unspecified temperature. Particles of Na_3_Zr_2_Si_2_PO_12_ were embedded in Na_3_PS_4_, yielding at 70% of the ceramic, a solid electrolyte with σ_100°C_ = 1.1 × 10^−3^ S/cm [262]. A composite electrolyte was prepared from PEO, NaClO_4_, Na_2_S, and P_2_S_5_ dissolved in acetonitrile, yielding Na_3_PS_4_/PEO/NaClO_4_ with σ_RT_ = 9.4 × 10^−5^ S/cm (only Na_3_PS_4_ σ_RT_ = 6.4 × 10^−5^ S/cm) at 4 wt.% of PEO and NaClO_4_, and tested in a Na/SnS_2_ battery [263]. A thin layer of PEO on Na_3_PS_4_ inhibited the detrimental chemical reaction of Na_3_PS_4_ with sodium and enabled more reversible sodium cycling, and greater fractions of PEO and NaClO_4_ resulted in a growing cell impedance because of a larger resistance of said coating.

A structural sodium-ion battery with a PEO-based composite electrolyte with NaClO_4_ as the salt and added Na_3_Zr_2_Si_2_PO_12_ showing σ_60°C_ = 1.02 × 10^−4^ S/cm and *t*_+_ = 0.44 kept 80% of its initial capacitance after 225 cycles [264].

The transition from liquid to solid electrolytes including hybrid systems for sodium-ion batteries has been surveyed; the acronym NIB (instead of the commonly accepted and used SIB) is certainly a highlight in this contribution in addition to many other inaccuracies [265], further aspects of this development are discussed in [34,266,267]. The knowledge of gel electrolytes for sodium-ion batteries has been surveyed in [268]. Electrolytes including composite ones based on NaCl for fuel cells and electrolyzers have been reviewed [269,270].

#### 3.1.2. Potassium and Potassium-Ion Batteries

As reported, KTFSI (actually this is potassium bis(trifluoromethanesulfonyl)imide) is not suitable for potassium-ion batteries because of its low melting point, making it mechanically unstable and thus not useful as a “separator” in addition to being the electrolyte [271]. Inorganic fillers Al_2_O_3_ and SiO_2_ were tried, and they increase the mechanical stability without negatively affecting ionic conductivity. Actually, mixtures or composites of KTFSI and PEO with added fillers were studied. Conductivities above the melting point of the composite (presumably not very helpful for practical application) were not affected by the mechanical filler; at actual operating temperatures, no numbers were provided. A full cell kept 93% of its initial capacitance after 100 cycles. A powder mixture of PEO, potassium β-alumina, and potassium trifluoromethanesulfonimide was pressed into a pellet and heated; in a separate procedure, these compounds were dissolved/suspended in DMF, yielding a membrane after solvent evaporation [272]. For a trilayer solid electrolyte, the latter liquid was poured on both sides of the pellet and dried. An interfacial resistance but no conductivity or performance in an actual cell were reported.

A composite of potassium polystyrenesulfonate and a polyoxovanadate (HK_5_[V_10_O_28_]·_10_H_2_O) was used as an electrolyte in a potassium/Prussian Blue battery [273].

A composite electrolyte of polypropylene carbonate with KFSI dissolved in ethylene carbonate/diethyl carbonate as the electrolyte and a nonwoven cellulose membrane as the filler and reinforcement has been prepared (σ_20°C_ = 1.36 × 10^−5^ S/cm) and tested in a potassium/3,4,9,10-perylene-tetracarboxylicacid-dianhydride battery [274]. The rapid capacity fading was attributed to the solubility of the active material in the positive electrode, but how the conclusion of a stable cycling performance could be reached remains mysterious.

A solid electrolyte for a potassium-ion battery was obtained by dissolving a powder of perfluorinated resin in DMF and adding some KPF_6_-containing electrolyte solution in ethylene carbonate and diethyl carbonate [275]. Using it in a complete cell yielded a sodium-ion battery with a capacitance decay of 0.26% per cycle during 200 cycles.

A solid electrolyte based on polyvinyl butyral with KCl and σ_30°C_ = 1.87 × 10^−5^ S/cm has been prepared and tested in a potassium-metal/iodine battery [276].

#### 3.1.3. Magnesium and Magnesium-Ion Batteries

##### Inorganic Electrolytes

Along the line reported by Liang [12], composites of Mg(NO_3_)_2_ and Al_2_O_3_ were prepared and their ionic conductivity examined [277]. With the composition 0.9Mg(NO_3_)_2_/0.1Al_2_O_3_, σ_RT_~10^−4^ S/cm was observed and attributed to the presence of MgO. A composite of Mg(BH_4_)_2_ with THF and MgO added for stabilization of the composition Mg(BH_4_)_2_·1.5THF-MgO(75 wt.%) yielded σ_70°C_~10^−4^ S/cm with *t*_+_ = 0.99 and was tested in a Mg/TiS_2_ battery [278]. A composite of Mg(BH_4_)_2_·CH_3_NH_2_BH_3_ with 75 wt.% MgO added for solidification of the initially viscous liquid showed σ_RT_~10^−5^ S/cm [279]. Borohydride−amide composites have been prepared by mechanochemical (ball milling) and thermal procedures (annealing with various protective gas atmospheres) and characterized in view of possible applications in magnesium batteries [280]. Prepared samples always contained Mg(BH_4_)_2_·(NH_2_) and some other phase, and conductivity reached σ_100°C_ = 3 × 10^−6^ S/cm, higher than earlier reported values of σ_150°C_ = 10^−6^ S/cm and σ_100°C_ = 10^−8^ S/cm [281]. The difference in reported conductivities—certainly less than the three orders of magnitude claimed in [280]—was attributed to some additional ionic phase.

Various cyclopropylamine borohydrides and some of their composites with Al_2_O_3_ have been prepared, characterized, and examined as solid electrolytes [282]. The composite electrolyte Mg(BH_4_)_2_·(CH_2_)_2_CHNH_2_·Al_2_O_3_ (50:50 by weight) had the highest conductivity σ_RT_ = 1.8 × 10^−5^ S/cm and *t*_+_ = 0.99999. A gel composite electrolyte composed of magnesium borohydride Mg(BH_4_)_2_, MgCl_2_, and polyethylene glycol (Figure 6) has demonstrated high thermal stability and ionic conductivity (σ_RT_ = 1.01 × 10^−4^ S/cm, *t*_+_ = 0.74) and good compatibility with a magnesium electrode [283]. Over 1000 cycles, 92.6% of the initial capacitance was retained.

Composite electrolytes based on silicate tetraethylene glycol hybrids with various magnesium salts have been prepared and characterized [284]. Conductivities around 10^−4^ to 10^−3^ S/cm were observed.

Composites of Mg(BH_4_)_2_ and various amounts of isopropylamine have been tested as magnesium-ion conductors [285,286]. The highest ionic conductivity of σ_45°C_ = 2.7 × 10^−4^ S/cm was found for the composition Mg(BH_4_)_2_·1.5(CH_3_)_2_CHNH_2_. Materials start to soften around 50 °C, and addition of 75 wt.% of MgO increased ionic conductivity as well as thermal stability, suggesting a contribution of hydrophobic interactions to increased conductivity. Mg(BH_4_)_2_·1.47CH_3_)_2_CHNH_2_ confined in mesoporous silica SBA-15 yielded σ_32°C_ = 9.1 × 10^−6^ S/cm [287].

##### PEO-Based Electrolytes

In a mechanochemical procedure, a mixture of PEO, Mg(ClO_4_)_2_, and EMIMFSI in acetone was ball-milled, which yielded a composite electrolyte after solvent evaporation with σ_RT_ = 0.5 × 10^−5^ S/cm [288].

To PEO with magnesium triflate as the salt, urea has been added, yielding a solid electrolyte with σ_?_ = 6.3 × 10^−5^ S/cm, with possible use in magnesium batteries suggested [289]. The increase in conductivity caused by added urea was attributed to faster segmental motion in PEO and lower viscosity of the medium. A composite electrolyte of PEO with MgBr_2_ as the salt and added starch nanocrystals was prepared with σ_RT_ = 7.8 × 10^−8^ S/cm [290]. Increased conductivity was attributed to additional ion transport channels formed by the starch nanocrystals. To a PEO-Mg(ClO_4_)_2_ solid electrolyte, nanochitosan was added as a filler, increasing the ionic conductivity by about two orders of magnitude around σ_60°C_ ≈ 10^−3^ S/cm with 10 wt.% salt and 10 wt.% filler [291]. A PEO-based composite electrolyte with Mg(OH)_2_ as a plasticizer also containing LiTFSI with σ_RT_ ≈ 1.6^−5^ S/cm has been reported [292]. A liquid (!) composite electrolyte of PEO with magnesium acetate and added nanoparticles of MgO showed σ_RT_ = 3.63 × 10^−3^ S/cm after γ-irradiation [293].

##### PVA-Based Electrolytes

A solid electrolyte of PVA with various amounts of Mg(ClO_4_)_2_ has been prepared and characterized, and an optimum conductivity σ_30°C_ ≈ 10^−4^ S/cm was recorded with PVA/salt composition 0.6/0.4 [294]. A PVA-based solid electrolyte with MgBr_2_ as a salt and phosphomolybdic acid (this is presumably the compound called phosphomolbidic acid throughout the report) and TEGDME has been prepared and tested in a Mg/TiO_2_ battery [295]. The highest observed ionic conductivity was σ_303 K_~10^−6^ S/cm, *t*_+_ = 0.4. A polymer electrolyte of PVA with MgBr_2_ and tetraethylene glycol dimethyl ether as a plasticizer was combined with Li_2_O, yielding a composite electrolyte with σ_RT_ ≈ 10^−5^ S/cm and *t*_+_ = 0.7 at 0.04 wt.% optimum filler content [296]. It was tested in a Mg/V_2_O_5_ battery.

A composite electrolyte of a blend of PVA and SN with a magnesium electrolyte has been characterized and tested in a Mg/TiO_2_ battery [297].

Structural and ion transport properties of a polymer blend of PVA and hydroxypropyl methylcellulose with Mg(NO_3_)_2_ as a salt (hardly as dopant as suggested in the report) have been studied [298]. At the optimum composition (PVA:HPMC = 0.4:0.6, 40 wt.% salt), σ_RT_ = 2.48 × 10^−4^ S/cm was measured, and said mixture had the lowest crystallinity based on XRD data, certainly supporting high ionic conductivity. Unfortunately, the mentioned conductivity value does not match tabulated data; this may be due to the use of NaNO_3_ for measurement of the tabulated data. A most uncommon use of transference number *t*_ion_ = 0.995 has been reported, and for the electrons, *t*_e_ = 0.005 has been listed. The clearly large value of *t*_ion_ is highlighted, but the cause of electronic conduction is not even mentioned. Presumably, parasitic currents are a cause. Back to standard practice, *t*_+_ = 0.247 was finally obtained. A primary Mg/MnO_2_ battery with this optimized composite electrolyte was assembled and discharged once.

##### PVDF-Based Electrolytes

To dissolved PVDF, MgBr_2_ was added as an electrolyte and some TEGDME as a plasticizer, yielding a composite electrolyte with σ_RT_ = 1.2 × 10^−6^ S/cm and *t*_+_ = 0.55, showing initially reversible magnesium plating both in a symmetric Mg/Mg and a battery-type Mg/graphene nanoplatelet cell [299]. XRD data show an almost complete loss of crystallinity of the polymer, and decreasing reversibility of magnesium plating was attributed to a growing “interfacial resistance”.

A membrane of a polymer blend of PVDF and polyurethane with MgO as a filler was soaked in a carbonate solvent-based electrolyte solution with Mg(ClO_4_) σ_60°C_ = 3.4 × 10^−6^ S/cm, yielding a gel composite electrolyte with σ_RT_ = 4.6 × 10^−3^ S/cm at the optimum filler content of 7 wt.% [300]. A mixture of PVDF-HFP, Mg(ClO_4_)_2_, and PYR_14_TFSI with 10 wt.% TiO_2_ as a filler has been made into a composite electrolyte with σ_RT_ = 0.16 × 10^−3^ S/cm [301]. In a symmetric Mg/Mg cell, highly reversible magnesium plating was observed.

A composite electrolyte from PVDF-HFP with Mg(ClO_4_)_2_ dissolved in a carbonate solvent mix with various amounts of fumed silica as a filler with σ_RT_~1.1 × 10^−2^ S/cm at optimum filler content 7 wt.% has been prepared and tested in a Mg/MoO_3_ battery [302]. Space-charge layers formed at the filler/polymer interfaces were suggested as the cause of the enhanced conductivity. The cell ran for more than 10 (!) cycles.

Into a blend of PVDF-HFP/polyvinyl acetate with Mg(ClO_4_)_2_ as the salt, geikeilite (MgTiO_3_) was added as an inorganic filler at various percentages [303]. The highest observed ionic conductivity σ_30°C_ ~ 5.80 × 10^−3^ S/cm and *t*_+_ = 0.34 were found at the optimum composition (6 wt.% of MgTiO_3_), and a complete cell retained 86% of its initial capacitance after 30 cycles.

With PVDF-HFP with magnesium triflate as a salt, and SN and some 1-ethyl-3-methylimidazolium trifluoromethanesulfonate for stabilization, a composite electrolyte with σ_26°C_ = 4 × 10^−3^ S/cm was obtained and tested in a Mg-C/MnO_2_ battery, showing substantial capacity fading within eight cycles (!) [304]. The different effects of active and passive fillers (see also [104]) on magnesium-ion conductivity of PVDF-HFP with Mg(Tf)_2_ in a mixture of ethylene and propylene carbonate with magnesium-containing and thus possibly actively participating compounds like MgO or MgAl_2_O_4_ and passive fillers like TiO_2_, Al_2_O_3_, or SiO_2_ have been studied [105]. Whether the absence of peaks in diffractograms clearly attributable to MgAl_2_O_4_ or Al_2_O_3_ proves complete dissolution appears to be questionable (see also [305]); more likely is a very fine distribution of the nanoparticles of the filler. With an optimum content of 30 wt.% Al_2_O_3_, σ_RT_ = 3.3 × 10^−4^ S/cm was found, and with 30 wt.% MgAl_2_O_4_, σ_RT_ = 4 × 10^−4^ S/cm. Addition of fillers resulted in significant increases in transference numbers to *t*_+_ = 0.52 in the former and *t*_+_ = 0.66 in the latter case. The remarkable difference between changes afforded by active vs. passive fillers was not resolved.

**Figure 21:**
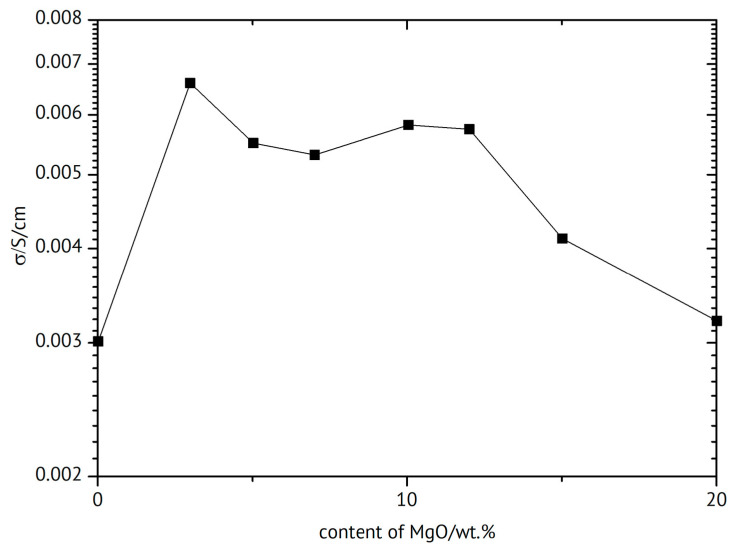
Ionic conductivity vs. MgO content (based on data in [306]).

A PVDF-HFP-based composite electrolyte with Mg(ClO_4_)_2_ dissolved in a mixture of ethylene carbonate and propylene carbonate (which stayed in the gel) and MgO as inorganic filler yielded σ_RT_~6 × 10^−3^ S/cm and *t*_+_ = 0.39 at optimum composition 10 wt.% filler and was tested in a Mg/V_2_O_5_ battery for ten (!) cycles [306]. Why the even higher conductivity at 3 wt.% filler was not considered the optimum (see Figure 21) was not addressed. Such observation of two maxima has been reported elsewhere [307,308,309,310]. The first maximum has been attributed to enhanced dissociation of ion aggregates and/or undissociated salt whereas the second one has been attributed to space charge effects discussed above [19,311,312,313]. At higher filler concentrations, the particles hinder ion movement and thus decrease conductivity [314].

##### Other Polymer-Based Electrolytes

Into a PEG-based electrolyte with magnesium acetate as salt various amounts of TiO_2_ have been added [315]. At optimum filler content 10 wt.% σ_RT_ = 5.01 × 10^−5^ S/cm was found, without filler it was σ_RT_ = 1.07 × 10^−6^ S/cm.

To a solid electrolyte of methyl cellulose and magnesium acetate, Co_3_O_4_ was added yielding a composite electrolyte with σ_RT_ = 5.93 × 10^−4^ S/cm at the optimum composition tested in a Mg/MnO_2_ battery [316]. For a magnesium/iodine battery, a PEG-based electrolyte with magnesium acetate as the salt and CeO_2_ as the inorganic filler has been prepared and tested [317]. Up to 15 wt.% of added filler, an increase in conductance to σ_60°C_ = 3.4 × 10^−6^ S/cm with t_+_ ≈ 0.97 was observed. Into a PEG-based electrolyte with Mg(NO_3_)_2_, TiO_2_ was added as an inorganic filler, providing a composite electrolyte with σ_RT_ = 1.06 × 10^−4^ S/cm and *t*_+_ = 0.98 at an optimum filler content of 10 wt.% tested in a Mg/I_2_ battery [318]. To a PEG-based polymer electrolyte with magnesium acetate, TiO_2_ was added as a ceramic filler, yielding a composite electrolyte with σ_RT_ = 5 × 10^−5^ S/cm [319]. To a chitosan-based polymer electrolyte with MgCl_2_, various amounts of V_2_O_5_ were added as inorganic filler, yielding a composite electrolyte tested in a Mg/MnO_2_ battery [320]. Added filler decreased the crystallinity of the composite, with 1 wt.% showing the most pronounced effect, which in turn increases ion mobility, i.e., conductivity. Indeed, conductivity (σ_RT_ = 1.4 × 10^−3^ S/cm and *t*_+_ = 0.96) was highest with this composition.

On a glass-fiber substrate, a glycerol α,α′-diallyl ether-3,6-dioxa-1,8-octanedithiol-based gel polymer electrolyte with σ_RT_ = 1.19 × 10^−4^ S/cm and *t*_+_ ≈ 0.704 was prepared and tested in a magnesium/Mo_6_S_8_ battery [321]. A composite electrolyte with nanosized MgO dispersed in poly(ethyl methacrylate) with MgTf_2_ and EMITFSI has been prepared [322]. Effects of various inorganic fillers on PEO-based composite electrolytes with Mg(CF_3_SO_3_)_2_ on ion transport properties have been studied, and the greatest increase in conductivity was found with 5 wt.% of MgO, where *t*_+_ = 0.38 was also the largest [308]. Performance improvements were largest with an active (Mg-containing) filler.

For a magnesium/sulfur battery, a gel electrolyte was prepared by a cross-linking reaction between Mg(BH_4_)_2_, Li(BH_4_), and polytetrahydrofurane reinforced with glass fiber separator (σ_0°C_ = 0.15 × 10^−3^ S/cm) and tested in magnesium batteries with various positive electrode materials [323]. With Mg/TiS_2_, a stable capacitance over 70 cycles was achieved.

Recent progress in magnesium battery research also addressing some composite electrolytes has been reviewed [324]. Progress and prospects of solid electrolytes for magnesium batteries are discussed in [325]. The effect of Na_2_SiO_3_ on the behavior of an AZ31B magnesium alloy anode in a mixed aqueous electrolyte solution with Na_2_SO_4_ and NaNO_3_ has been examined [326].

#### 3.1.4. Calcium and Calcium-Ion Batteries

Various urea calciumtetrahydridoborate compositions have been synthesized and characterized [327]. The highest ionic conductivity was found for the composition Ca(BH_4_)_2_·3.30CO(NH_2_)_2_ at σ_RT_ = 2.46 × 10^−7^ S/cm with *t*_+_ = 0.997.

#### 3.1.5. Zinc and Zinc-Ion Batteries

Rechargeable zinc/MnO_2_ batteries (the approved acronym is RAM for rechargeable alkaline manganese, not eAZMB as suggested by the authors in [328]) have been pursued for some time but discontinued, mostly because of problems related to the zinc electrode [329,330]. Based on advances with reversible zinc electrodes, there may be a renaissance. As an electrolyte for such systems, a composite of a gelatin hydrogel with fumed silica has been studied [328]. The addition of SiO_2_ increased porosity and ionic conductivity, and zinc corrosion was reduced and zinc deposition proceeded more uniformly; 10%wt. of SiO_2_ was the optimum fraction. Hierarchically porous ZIF-67 filled with PVP combined with nanocellulose yielded a composite with σ_RT_ = 4.31 × 10^−3^ S/cm, used as separator in an aqueous zinc battery [331]. To cellulose acetate with NH_4_BF_4_, SiO_2_ was added as a filler, yielding a composite electrolyte with σ_RT_ = 7.89 × 10^−3^ S/cm at 3 wt.% filler addition, tested in a Zn/MnO_2_ battery [332]. Ammonium ions were suggested as mobile charge carriers, but why the device is called a proton battery remains mysterious. Into an anhydrous PVDF-HFP-based gel polymer electrolyte with an ionic liquid with conductivity σ_RT_ = 24.32 × 10^−3^ S/cm and *t*_+_ = 0.42 for a zinc/MnO_2_ battery, copper ions were immobilized in PAA (nowhere spelled out, most likely poly acrylic acid) for accelerated zinc ion movement and deposition [333]. The cell kept its capacitance for 1000 cycles.

The 2 M ZnSO_4_ electrolyte solution for an aqueous electrolyte has been modified by the addition of various tetraazacyclotetradecane compounds at concentrations ranging from 10 to 100 mM, resulting in impressively increased cycle numbers of the zinc electrode [334]. Modification of the coordinative environment of the zinc ions was suggested as the reason for suppression of dendritic zinc deposition. Zinc ion transport in a gel electrolyte prepared from poly(vinyl chloride) and poly(ethyl methacrylate) with 1-ethyl-3-methylimidazolium bis(trifluoromethylsulfonyl)imide (EMIMTFSI) as the gelling agent and Zn(OTf)_2_ as the salt modified with SnO_2_ nanoparticles yielded an optimum value σ_RT_ = 4.92 × 10^−4^ S/cm at 3 wt.% filler [335]. A very similar approach with fumed silica nanoparticles instead of tin oxide providing σ_RT_ = 6.71 × 10^−4^ S/cm has been reported by these authors [336], with ZrO_2_ yielding σ_RT_ = 3.63 × 10^−4^ S/cm at a 3 wt.% filler content [337] or with PVDF-HFP instead yielding σ_RT_ = 4.6 × 10^−4^ S/cm at a 7 wt.% filler content [338].

To PVDF-HFP (abbreviated quite unconventionally and thus confusing PVHF) dissolved in acetone EMIMBF_4_ (again confusingly abbreviated as EMIBF_4_ except for the common acronym in the title), Zn(OTF)_2_ and Ti_3_C_2_T_x_ were added (the meaning of “suspended above the polymer solution” remains mysterious), yielding a composite electrolyte (σ_60°C_ = 2.11 × 10^−3^ S/cm and σ_20°C_ = 1.75 × 10^−3^ S/cm), which was tested in a zinc/NVP battery [339]. In addition to 80% capacitance retention after 1000 cycles, “controlled self-discharge” appears to be the most prominent feature of this battery.

**Figure 22:**
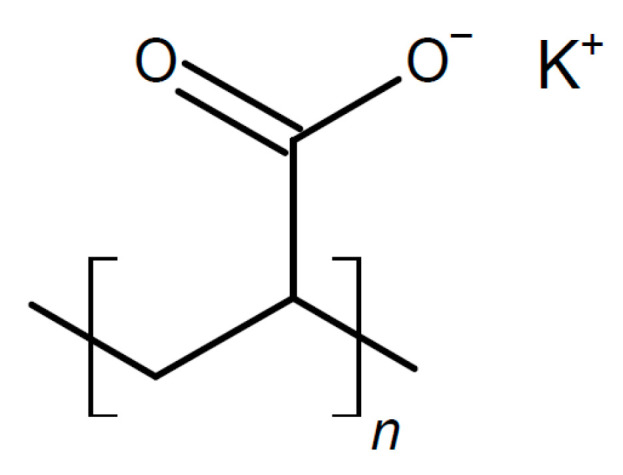
Molecular structures of potassium polyacrylate.

A liquid water-in-polymer salt electrolyte has been prepared from potassium polyacrylate (see Figure 22) and Zn(TFSI)_2_ and tested in a zinc/lignin battery, retaining 80% of its initial capacitance after 8000 cycles [340].

##### PVA-Based Electrolytes

A PVA-based gel electrolyte with ZnSO_4_ was made into a composite by adding ZnO nanopowder, increasing ionic conductivity from σ_RT_ = 2 × 10^−4^ S/cm to σ_RT_ = 1.3 × 10^−3^ S/cm [341]. A PVA-based gel with KOH as the electrolyte and added functionalized carbon nanotubes (CNTs) (graphene oxide is nowhere found in the experimental part) showed σ_RT_ = 6.2 × 10^−2^ S/cm and *t*_ion_ = 0.9, suggesting substantial electronic conduction, which is even greater with *t*_ion_ = 0.55 without any filler, leaving the question of what provides electronic conduction in the filler-free gel electrolyte (for the GO-containing version, σ_RT_ = 6.9 × 10^−2^ S/cm and *t*_ion_ = 0.87) [342]. How the data suggest that hydroxyl ions are the charge carriers provided by KOH remains unclear. The electrolyte was tested in a zinc/Ag_2_O battery.

A PVA-based composite gel with added sulfonated cellulose and sepiolite was soaked in a 2 M ZnSO_4_ solution (σ_RT_ = 2.2 × 10^−2^ S/cm) and used in a zinc/I_2_ battery, showing 83% capacity retention after 10,000 cycles [343].

Hydroxyethyl cellulose (HEC) and montmorillonite (MMT) were added into PVA, yielding a composite electrolyte after soaking in an aqueous solution of 2 M ZnSO_4_ and 0.1 M MnSO_4_ with σ_RT_ = 24.6 × 10^−3^ S/cm at the optimum composition (3 wt.% HEC and 2 wt.% MMT) [344]. A zinc/MnO_2_ battery with this electrolyte kept 91% of its initial capacitance after 450 cycles. A PVA-based gel electrolyte with Zn(CF_3_SO_3_) was combined with Prussian Blue for an enhanced zinc ion transference number and better water retention, yielding a composite electrolyte with σ_RT_ = 16.3 × 10^−3^ S/cm and *t*_+_ = 0.63 for a zinc/ammonium vanadate (NVO) battery [345]. PVA modified with an inorganic filler ZnO, poly(*m*-phenylene isophthalamide) as reinforcement, glycerol as antifreeze agent, and Zn(CF_3_SO_3_)_2_ had σ_20°C_ = 9.7 × 10^−3^ S/cm and was used as a composite electrolyte in a zinc/MnO_2_ battery, losing 10% of its initial capacitance after 1200 cycles [346]. A PVA-based gel reinforced with nanofibers of polyurethane-poly(*m*-phenylene isophthalamide) used as a solid electrolyte in a Zn/MnO_2_ battery enabled operation for 6500 h [347]. A PVA-based gel electrolyte with Zn(OTf)_2_ was combined with aramid nanofibers into a composite electrolyte with σ_RT_ = 42.2 × 10^−3^ S/cm and *t*_+_ = 0.78 and tested in a zinc/polyaniline battery, keeping 78% of its initial capacitance after 9100 cycles [348].

A membrane of electrospun poly(*m*-phenylene isophthalamide) fibers was decorated with ZnO nanorods and filled with PVA, yielding a zinc ion-conducting solid electrolyte (σ_RT_ = 18.3 × 10^−3^ S/cm) for a Zn/MnO_2_ battery running for 1000 cycles [349]. High conduction was attributed to transport channels along the ZnO nanorods. A mechanically stable and recyclable hydrogel P_3_B_2_Z_x_ has been prepared by cross-linking PVA (P) with borate (borax B) and ZnCl_2_ (Z) as the salt [350]. Depending on the salt content, conductivity varied from σ_RT_ = 3.5 × 10^−3^ S/cm to σ_RT_ = 33.8 × 10^−3^ S/cm when going from P_3_B_2_Z_1_ to P_3_B_2_Z_2.5_. A full battery kept 77% of its capacity after 1500 cycles.

##### PAM-Based Electrolytes

**Figure 23:**
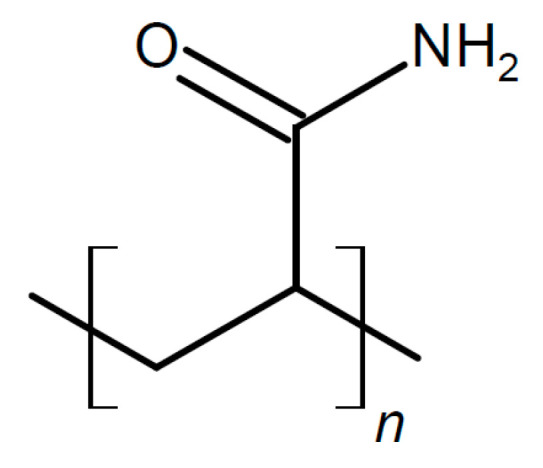
Molecular structures of polyacrylamide PAM.

**Figure 24:**
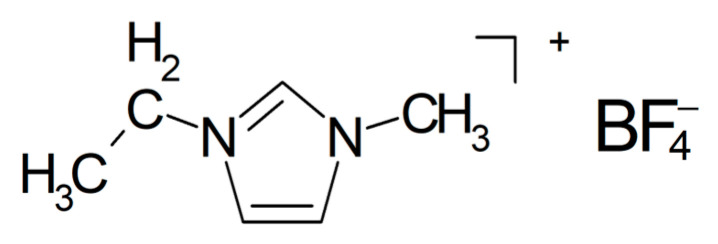
Molecular structures of 1-ethyl-3-methylimidazolium tetrafluoroborate EMIMBF_4_.

A gel based on polyacrylamide PAM (see Figure 23) with ZnSO_4_ as salt, EMIMBF_4_ (see Figure 24) (the salt is certainly no co-solvent as claimed in the report), and a deep eutectic Zn(ClO_4_)/EG with *t*_+_ = 0.915 was tested in a zinc/VO_2_ cell, keeping its initial capacitance for 200 cycles [351]. The claimed exceptional ionic conductivity was unfortunately not reported in numerical terms.

A composite solid electrolyte prepared from PAM and an unspecified salt, presumably ZnSO_4_, with coffee silverskin cellulose added as a filler (why and how the resulting material was combined with ZnSO_4_ remains unclear) with σ_RT_ = 36.51 × 10^−3^ S/cm (without filler, σ_RT_ = 25.85 × 10^−3^ S/cm) was used in a zinc/MnO_2_ battery [352]. PAM was prepared in an aqueous solution also containing 1 M ZnSO_4_, yielding a polymer electrolyte into which a small amount of graphitic C_3_N_4_ was added [353]. This resulted in less corrosion of the zinc electrode and a much smoother zinc deposition, the zinc ion transference number increased from *t*_+_ = 0.383 to *t*_+_ = 0.631, the composite electrolyte enabled a zinc/carbon cell with 87% capacitance retention after 1000 cycles, and without the added g-C_3_N_4_, only 55% was retained. PAM was polymerized from a monomer-containing solution with carboxymethylcellulose and dissolved ZnSO_4_, and dispersed graphene oxide (not graphene as claimed in the title of the report) [354]. The composite electrolyte showed σ_RT_ = 45.5 × 10^−3^ S/cm, and a complete zinc/NVO battery kept 82% of its initial capacitance after 800 cycles. A hydrogel electrolyte based on a polymer blend of PAM and diacetone acrylamide with dickite (Al_2_Si_2_O_5_(OH)_4_) as a filler was soaked in an aqueous solution of 2 M ZnSO_4_ and 0.1 M MnSO_4_, yielding a composite electrolyte with σ_RT_ = 20.7 × 10^−3^ S/cm at the optimum composition [355]. Smooth zinc deposition was attributed to the added dickite, resulting in a stable capacitance of a Zn/MnO_2_ battery over at least 700 cycles.

A PAM-gel with hemp-derived cellulose fibers was soaked in a solution of 2 M ZnSO_4_ and 0.5 M MnSO_4_, affording a composite electrolyte with σ_RT_ = 59.6 × 10^−3^ S/cm at 1 wt.% fibers and was tested in a zinc/CoMn_2_O_4_ battery, keeping 56% of its initial capacitance after 500 cycles [356].

A PAM-based gel reinforced with a lamellar–porous polyimide covalent organic framework and ZnSO_4_ as an electrolyte with σ_RT_ = 21.7 × 10^−3^ S/cm (with reinforcement σ_RT_ = 10.9 × 10^−3^ S/cm) and *t*_+_ = 0.74 has been tested in a zinc/NVO battery with 96% capacitance retention after 1500 cycles [357]. For a cathode-less zinc/manganese fiber battery, Zn^2+^-containing neutral, weakly acidic, and acidic PAM-based gel electrolytes were prepared [358]. The weakly acidic one was used between the electrodes, and the polymer-coated zinc wires were immersed into the neutral one, whereas the acidic one was placed close to the cathode of the cathode-less (!) device. The battery kept 76% of its initial capacitance after 2700 cycles. A hydrogel prepared from agar and PAM (5:1) with ZnSO_4_ dissolved in ethylene glycol as the electrolyte with *t*_+_ = 0.339 has been tested in a zinc/V_2_O_5_ battery [359].

A polymer blend of PAM and CMC modified and made into a composite electrolyte from an aqueous solution of 2 M ZnSO_4_ and 0.1 M MnO_4_ with 2 g/L sodium dodecyl sulfate (SDS) (σ_RT_ = 28.05 × 10^−3^ S/cm; without SDS, σ_RT_ = 26.07 × 10^−3^ S/cm; PAM only, σ_RT_ = 9.15 × 10^−3^ S/cm; *t*_+_ in this sequence 0.82; 0.57; 0.27) was tested in a zinc/MnO_2_ battery, keeping 66% of its initial capacitance after 350 cycles [360]. Dominant zinc deposition on the 002-plane was attributed to the added SDS, which also reduces parasitic hydrogen evolution.

PAM was polymerized inside a network of agarose and CMC in an aqueous solution also containing 1 M ZnSO_4_ and 0.1 M MnSO_4_, yielding a composite gel electrolyte with σ_RT_ = 23.1 × 10^−3^ S/cm at the optimum composition and was subsequently tested in a zinc/MnO2 battery, keeping 80% of its initial capacitance after 800 cycles [361].

##### Biopolymer-Based Electrolytes

A biocompatible gel of a blend of agarose and sodium alginate soaked in an aqueous solution of 2 M ZnSO_4_ and 0.1 M MnSO_4_, yielding an electrolyte with σ_RT_ = 25.05 × 10^−3^ S/cm and *t*_+_ = 0.75, was tested in a flexible zinc/MnO_2_ battery, which kept 83% of its initial capacitance after 800 cycles [362]. For stabilization of the water in a sodium alginate gel electrolyte, the disaccharide trehalose was added, yielding σ_RT_ = 12.4 × 10^−3^ S/cm; it was tested successfully in a zinc/polyaniline battery, with 70.4% capacitance retention after 500 cycles [363]. A sodium alginate hydrogel electrolyte modified with urea was used in a biocompatible printable flexible zinc-ion battery [364]. Urea regulated zinc nucleation kinetics. A cotton pad treated with sodium alginate and soaked with aqueous zinc acetate solution provided σ_RT_ = 1.96 × 10^−3^ S/cm when treated with 1 wt.% alginate solution, and this separator provided the best zinc cycling [365]. A composite hydrogel of sodium alginate and carrageenan with a zinc-ion transference number *t*_+_ = 0.58 and σ_RT_ = 5.89 × 10^−2^ S/cm has been tested in a zinc/NH_4_V_4_O_10_ battery for 600 cycles [366]. A sodium alginate-based gel electrolyte with added nano-SiO_2_ has been reported [367]. When used in a Zn/MnO_2_ battery with an aqueous neutral ZnSO_4_ gel electrolyte also containing some MnSO_4_ and Cs_2_SO_4_, the added filler increased the available cycle number from 1000 to 1800 with 78% capacitance retention in the latter case. A polymer blend of sodium alginate and PAM (presumably this is meant, with the acronym PAAM nowhere explained in the report) with ZnCl_2_ as the electrolyte and graphene oxide as the filler with σ_RT_ = 12.12 × 10^−3^ S/cm was used as a composite electrolyte in a zinc/LiFePO_4_ battery, with 63% capacitance retention after 500 cycles [368]. Into a hydrogel of cross-linked sodium alginate and acrylamide, PVP-coated nanosheets of alumina were added; the obtained composite electrolyte membrane was soaked in an aqueous solution of zinc trifluoromethanesulfonate (Zn(OTf)), providing σ_RT_ = 17 × 10^−3^ S/cm [369]. A complete zinc/PVO (vanadium pentoxide with poly(3,4-ethylenedioxythiophene)) battery kept 83% of its initial capacitance over 1000 cycles. A blend of PVA, sodium alginate, and polyvinyl pyrrolidone gel polymer electrolyte showed σ_RT_ = 9.46 × 10^−3^ S/cm (presumably due to the immersion of the gel in a mixed solution of ZnSO_4_ and Li_2_SO_4_), and when tested in a zinc/LiFePO_4_ battery, showing 68% capacitance retention after 1000 cycles [370]. A sodium alginate electrolyte with ZnSO_4_ as the salt has been modified by adding tannic acid, yielding a composite electrolyte (σ_RT_ = 24 × 10^−3^ S/cm; without tannic acid, σ_RT_ = 20 × 10^−3^ S/cm), tested in a zinc/NH_4_V_4_O_10_ battery with 97% capacitance retention after 200 cycles [371]. An improved performance in terms of more reversible zinc plating achieved by added tannic acid was stated.

Chitosan mixed with NH_4_NO_3_ and ethylene carbonate as a plasticizer yielded a composite electrolyte used in a zinc/MnO_2_ battery [372].

From lignin and fumed silica, with the addition of an aqueous electrolyte solution of 2 M ZnSO_4_ and 0.2 M MnSO_4_, a composite gel electrode was prepared, and it was tested in a zinc/MnO_2_ battery with an almost constant capacity during 3000 cycles [373]. This performance suggests strong dendrite formation suppression, low zinc corrosion, and less byproduct formation, as confirmed by analytical verification.

To a mixture of carboxy methylcellulose and poly(*N*-isopropylacrylamide) with ethylene carbonate as plasticizer and zinc triflate as a salt, Ti_3_AlC_2_ (MAX) was added, yielding a composite electrolyte for a Zn/NVO battery [374]. Strong interaction between MAX and the triflate anion was suggested as a reason for the unspecified high ionic conductivity and transference number. A hydrogel electrolyte of sodium CMC and sodium alginate soaked in a 1.5 M ZnSO_4_ and 0.1 M MnSO_4_ solution was tested in a zinc/MnO_2_ battery, keeping 48% of its initial capacitance after 500 cycles [375].

A hydrogel of gelatin and *γ*-polyglutamic acid soaked in an aqueous solution of 2.5 M ZnSO_4_ and 0.3 M MnSO_4_ yielded a composite electrolyte with σ_RT_ = 12.6 × 10^−3^ S/cm, which was tested in a zinc/MnO_2_ battery, keeping 70% of its initial capacitance after 1000 cycles [376]. The added *γ*-polyglutamic was identified as the reason for smooth zinc deposition, less corrosion, and byproduct formation.

##### Miscellaneous Electrolytes

A composite of 70 parts P_2_O_5_·5H_2_O and 30 parts of a mix of 92 parts SiO_2_ and 8 parts of Al_2_O_3_ served as proton-conducting solid electrolyte in a battery with a negative zinc/zinc sulfate and a positive MnO_2_/graphite electrode [377]. A composite electrolyte based on a non-woven fabric soaked with a slurry of PTFE and Mg(OH)_2_ particles was used in a zinc/carbon battery (also called a hybrid capacitor in the report) [378].

A hydrogel prepared from polyethylene-g-poly(acrylic acid) with added organo-montmorillonite was used with ZnCl_2_ as a salt as electrolyte in a Zn/carbon battery hydrogel [379]. Comparable data on performance and stability were not provided.

A porous sponge of cross-linked bacterial cellulose was filled with a hydrogel of poly(sodium 4-styrenesulfonate)/poly(dimethyl diallyl ammonium chloride), which was “loaded” either with a solution of 6 M KOH or 3 M KI, yielding σ_RT,KOH_ = 42.5 × 10^−3^ S/cm and σ_RT,KI_ = 29.0 × 10^−3^ S/cm [380]. The cellulose sponge without gel filling provided higher conductivities as expected.

A UV-curable gel electrolyte processable by ink-jet printing for a conformal zinc-ion battery has been reported [381].

Addition of CuCl_2_ and poly(N-diallyldimethylammonium chloride) to an aqueous ZnSO_4_ electrolyte solution resulted in a smoother zinc deposition [50]. For a zinc/iodine battery, the zinc electrode was coated with a zinc-ion selective electrolyte layer of poly(ether-*block*-amide) modified with graphene oxide [382]. The zinc ion transference number was *t*_+_ = 0.77; more than 36,000 cycles with 95.5% of the initial capacitance retained were recorded.

A mixture of several compounds (called for unknown reasons a composite) has been added to a presumably aqueous electrolyte solution for improved performance of a zinc-ion battery [383]. A membrane prepared from cellulose fibers surface-modified with tannic acid providing phenolic hydroxyl groups has been suggested for use in aqueous zinc-ion batteries [384]. By supporting a uniform distribution of zinc ions, high reversibility of the zinc electrode in various electrolyte solutions was enabled, yielding 83% capacitance retention after 1000 cycles.

Hydrogel-based electrolytes suggested for zinc-ion batteries show some deterioration during operation, attributed to densification resulting in poor electrolyte/electrode contact; a modified hydrogel showing stronger adhesion has been reported [385].

The separator of a zinc/LiMn_2_O_4_ battery has been soaked with a mixed electrolyte solution of ZnSO_4_ and Li_2_SO_4_, which was subsequently gelled with fumed silica [386]. Gelling of the liquid electrolyte solution resulted in a substantial improvement of cycling performance, a well-known observation with lead-acid batteries.

##### Zinc–Air Batteries

A hydroxyl-ion-conducting composite based on a Bakelite-type polymer with covalently attached viologen units coated onto filter paper was tested as an electrolyte/separator for a zinc/air battery [387]. Another strategy towards an improved interface by surface polymerization on the air electrode has been suggested [388]. In addition to proper zinc deposition, dehydration of water-containing electrolytes is a further challenge for zinc/air batteries.

A sodium polyacrylate gel electrolyte modified with graphene oxide or graphene oxide nanoribbons and cellulose nanofibers soaked in a solution of 6 M KOH and 0.2 M zinc acetate, yielding a composite electrolyte with σ_RT_ = 268 × 10^−3^ S/cm (with GO only σ_RT_ = 188 × 10^−3^ S/cm), was tested in a flexible zinc–air battery [389]. GO and GO nanoribbons were considered as plasticizers without further explanation and not supported by the displayed XRD results.

A PAA-based gel electrolyte with Al_2_O_3_ added as filler yielded a composite electrolyte after soaking in an aqueous solution of 6 M KOH and 0.2 M zinc acetate [390]. At the optimum filler content (30 wt.%) σ_RT_ = 186 × 10^−3^ S/cm, unfortunately, at this composition, water retention was slightly inferior to the composite with 20 wt.%. Presumably this property also enabled the slightly longer lifetime of a battery with this electrolyte composition.

A sodium CMC-PAA blend with KOH as a salt has been prepared, characterized, and suggested as an electrolyte for a zinc/air battery [391]. A ternary composite hydrogel of CMC, polyacrylamide, and graphene oxide has been proposed as a remedy [392]. A solid composite electrolyte based on CMC blended with either PVA or PAA yielded σ_RT_ = 231 × 10^−3^ S/cm at a composition of CMC:PAA 1:2 when the solid was soaked in aqueous KOH solution; it was proposed for use in zinc/air batteries [393]. A hydrogel prepared from CMC, PAM, and cellulose nanofibers (the acronym CNF used in this report appears to stand for carbon nanofibers in the rest of the world), with KOH or KCl as electrolytes, with σ_RT_ = 290 × 10^−3^ S/cm in the former case (and σ_RT_ = 163 × 10^−3^ S/cm without the cellulose nanofibers) and σ_RT_ = 171 × 10^−3^ S/cm in the latter case, was tested in a zinc/air cell [394].

A composite gel electrolyte of PVA with embedded cross-aligned polyacrylic nanofibers soaked in aqueous KOH solution provided σ_RT_ = 235 × 10^−3^ S/cm and has been proposed for use in zinc/air batteries [395]. Into a porous PVA gel (using PEG as pore-forming agent), various amounts of silica were added, yielding after soaking in 6 M KOH a composite electrolyte, tested in a flexible zinc/air battery [396]. At the optimum silica content, σ_RT_ = 57.3 × 10^−3^ S/cm was found, and this value decreased rapidly within 22 h; nevertheless, the best performance of a battery with this electrolyte was claimed. A composite electrolyte of PVA functionalized with quaternary ammonium groups combined with chitosan and MoS_2_ has been prepared, which (not mentioned in the experimental part) was finally soaked in an aqueous KOH-solution [397]. Addition of chitosan caused a higher uptake of the KOH solution. At 0.5 wt.% MoS_2_, σ_RT_ = 87.3 × 10^−3^ S/cm, where presumably MoS_2_ enhanced hydroxide ion mobility and increased longevity. A test in a zinc/air battery ran for 465 cycles. A gel of PVA with carboxylated nanocellulose fibrils soaked in aqueous 6 M KOH afforded a composite electrolyte with σ_RT_ = 312 × 10^−3^ S/cm, tested in a zinc/air battery [398]. A PVA gel reinforced with electrospun polyetherimide nanofibers apparently swollen in a KOH electrolyte, yielding a composite electrolyte with σ_RT_ = 13 × 10^−3^ S/cm, was tested in a rechargeable zinc/air battery [399]. Inhibition of zincate crossover deemed essential for a secondary zinc/air battery was observed. A near-neutral gel polymer composite electrolyte of PVA with functionalized MXene prepared in an aqueous KOH solution with σ_RT_ = 58.5 × 10^−3^ S/cm and a good water retention caused by the functionalized filler was tested in a rechargeable zinc/air battery [400]. In a somewhat mysterious procedure, a PVA-based gel with added Lyocell fibers was made into a composite electrolyte and suggested as replacement of an unspecified “commercialized composite separator” for a primary zinc/air battery [401]. A gel of PVA reinforced with epoxy resin as a composite electrolyte (σ_RT_ = 77.6 × 10^−3^ S/cm) was cast on the negative electrode composite and subsequently soaked in aqueous KOH solution, establishing a tight contact between electrolyte and electrode in a zinc/air battery [402].

A PVA gel with ZnSO_4_ and MnSO_4_ as salts was reinforced with bacterial cellulose fibers, yielding a composite electrolyte with σ_RT_ = 80.8 × 10^−3^ S/cm at an optimum filler content of 6 wt.% [403]. In a flexible secondary zinc–air battery, 650 cycles were run with only a minute deterioration of performance. The composite electrode could handle internal pressure changes during cycling, kept hydration water well, and suppressed dendrite formation and corrosion.

A PVA gel was modified with recycled polyurethane particles, onto which flexible polyurethane chains had been grafted and polydopamine had been deposited, yielding a flexible composite electrolyte with improved mechanical properties and ionic conductivity after soaking in a 6 M KOH solution with 0.2 M zinc acetate [404]. The composite showed σ_RT_ = 175 × 10^−3^ S/cm, plain PVA only σ_RT_ = 108 × 10^−3^ S/cm, and the composite with polyurethane particles only 131 × 10^−3^ S/cm, suggesting a beneficial effect of the grafted chains and the deposited amine, whereas the extended cell lifetime and improved zinc deposition also indicate an improved zinc ion distribution.

**Figure 25:**
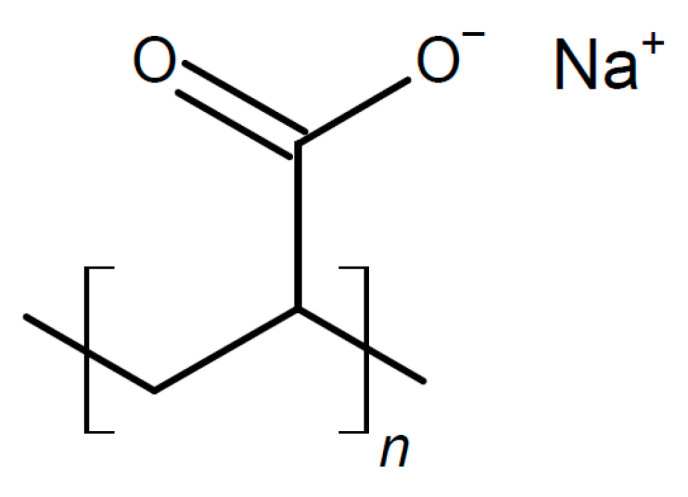
Molecular structures of sodium polyacrylate.

Into a mixed organic network of sodium polyacrylate (see Figure 25) and PVP, nanoparticles of TiO_2_(NH_2_) were added, yielding a gel polymer electrolyte for a zinc/air battery with enhanced water retention properties, capable of suppressing zinc dendrite formation [405]. Addition of the filler increased the electrolyte solution uptake to 13.8 times the solid’s weight and σ_RT_ = 272 × 10^−3^ S/cm.

The influence of the size of SiO_2_ nanospheres on the ionic conductivity of a composite with a blend of PVA and PAA, yielding a composite claimed to be porous (presumably the composite, not the polymer blend) and capable of reducing dendrite formation in a zinc/air battery, has been studied [406]. To a polymer blend of PVA and PAA, cellulose was added, and the composite was soaked in a solution of KOH and zinc acetate, yielding a composite electrolyte with σ_RT_ = 123 × 10^−3^ S/cm, tested in a secondary zinc/air battery [407].

A composite electrolyte of sodium polyacrylate with added nanocellulose and graphene oxide and σ_RT_ = 178 × 10^−3^ S/cm was used in a zinc/air battery after soaking in a mixed aqueous electrolyte solution of zinc acetate and potassium hydroxide [408]. For a rechargeable zinc/air battery, a hydrogel of polyacrylamide-carboxymethyl chitosan-ethylene glycol polymer blend was soaked in aqueous KOH/zinc acetate solution with σ_RT_ = 278 × 10^−3^ S/cm (which declined quickly within hours) and tested in a battery for 54 h [409]. A composite of PAM with various amounts of mesoporous SiO_2_ was soaked in aqueous KOH, yielding a composite electrolyte with σ_RT_ = 337 × 10^−3^ S/cm at an optimum filler content of 5 wt.% (this is presumably misleadingly called 5 wt.% mPAM in the report) and tested in a zinc/air battery [410]. To a PAM-based hydrogel with Zn(OTf)_2_ as a salt, fumed silica was added for enhanced water retention as a solid electrolyte in a zinc/air battery [411].

The influence of silica nanoparticles and PEG (see Figure 5) added into a polymer blend of PAM and CMC immersed presumably in an electrolyte solution of 4 M KOH or 4 M KOH + 2 M KI in water, yielding a composite electrolyte, has been studied [412]. Addition of KI resulted in a major increase in ionic conductivity from σ_RT_ = 135 × 10^−3^ S/cm to σ_RT_ = 312 × 10^−3^ S/cm, where the use of 2.8 M NH_4_Cl + 0.6 M ZnCl_2_ or 2.8 M KCl yielded σ_RT_ = 161 × 10^−3^ S/cm and σ_RT_ = 69 × 10^−3^ S/cm, respectively. The claimed benefits of the added PEG and silica are impossible to appreciate in the absence of comparable data; the mitigation of the “carbonate problem” expected with an alkaline electrolyte by use of a near-neutral electrolyte may possibly balance the somewhat worse overall performance.

A composite electrolyte of PEO, PVA, and KOH with a glass fiber mat has been prepared, characterized, and tested in a primary zinc/air battery [413]. With the optimum polymer mixture (50:50), σ_30°C_ = 47.5 × 10^−3^ S/cm was observed.

A composite electrolyte based on surface-functionalized nonwoven polypropylene/polyethylene fabric was coated with PVA and with additional acrylic acid monomer, yielding a composite electrolyte with σ_RT_ = 0.16. 0.21 × 10^−3^ S/cm and *t*_−_ = 0.83. 0.91 for a zinc–air battery (called a metal–air fuel cell) [414]. A composite of PVA and up to 2.5 wt.% mesoporous silica MCM-41 soaked in an aqueous solution of KOH (σ_RT_ = 380 × 10^−3^ S/cm; without silica, σ_RT_ = 340 × 10^−3^ S/cm) was tested in a zinc/air battery running for 145 cycles [415]. Without the filler, 163 cycles were achieved. To overcome the claimed insufficient stability of PVA-KOH electrolytes, a composite of sulfonated cassava starch, nano-attapulgite, and polyacrylamide finally ion-exchanged with aqueous KOH was prepared, enabling a flexible zinc/air battery running for 450 h [416].

A polymer blend of PAM and CMC was reinforced with hyaluronic acid (HA), yielding a gel, which was soaked in an electrolyte solution of unspecified composition, later stated as aqueous 6 M KOH with 0.2 M zinc acetate, yielding a composite electrolyte with σ_RT_ = 321 × 10^−3^ S/cm, or without HA, only σ_RT_ = 180 × 10^−3^ S/cm [417]. The displayed “power density curves of the electrolyte” actually refer to a full zinc/air battery. A gel composed of guar gum and sodium alginate soaked in a water/ethylene glycol solution of 2 M ZnSO_4_ as a salt with σ_RT_ = 25.37 × 10^−3^ S/cm was tested as a composite electrolyte in a zinc/MnO_2_ battery, keeping 91% of its initial capacitance after 1000 cycles [418]. Further general considerations on zinc/air battery development have been collected [419].

A wider overview on prospects of anion exchange membranes known from fuel cells and electrolyzers in metal-ion batteries is available [420].

##### Electrolytes for Structural Batteries

**Figure 26:**
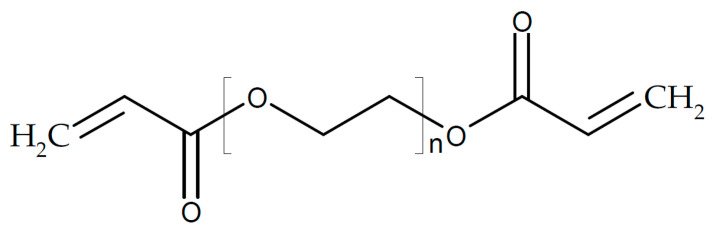
Molecular structures of poly(ethylene glycol) diacrylate.

A composite electrolyte of poly(ethylene glycol) diacrylate (Figure 26) reinforced with glass fibers for structural carbon fiber zinc-ion batteries has been described; it enabled a zinc-ion battery to run stably for 1000 cycles [421].

A “biomimetic” electrolyte of PEO with Zn(CF_3_SO_3_)_2_ as a salt and with branched aramid nanofibers (σ_RT_ = 2.5 × 10^−5^ S/cm) as a filler and reinforcement has been tested in a structural zinc/MnO_2_ battery, showing 96% capacitance retention after 50 cycles [422].

To a glass-fiber reinforced PVDF-HFP, an unspecified substance KL and KL-Z, presumably kaolin, was added, yielding a structural electrolyte with Zn(CF_3_SO_3_)_2_ as a salt (σ_RT_ = 0.44 × 10^−3^ S/cm and *t*_+_ = 0.78), subsequently tested in a zinc/ammonium vanadate battery [423]. The battery kept 95% of its initial capacitance after 500 cycles.

An epoxy-based solid electrolyte with EMIMTFSI and Zn(TFSI)_2_ was tested in a structural zinc/NH_4_V_4_O_10_ battery [424]. With the optimum composition, the battery barely showed any degradation after 140 cycles.

A biomimetic composite electrolyte based on quaternary ammonium-functionalized PVA deposited into an Aramid nanofiber membrane (σ_RT_ = 61 × 10^−3^ S/cm) for use in a structural zinc–air battery for robotic devices has been developed [425].

#### 3.1.6. Aluminum and Aluminum-Ion Batteries

An aqueous electrolyte solution containing a mixture of Al(OTF)_3_, HOTF, and Zn(OTF)_2_ for a battery with a negative Al-Zn electrode and a positive Al_x_MnO_2_·*n*H_2_O has been developed [139]. The battery ran for 100 cycles.

Addition of 3 wt.% of cellulose nanofibrils (the acronym CNF appears to be a confusing assignment) to polyacrylic acid with aqueous KOH solution as the gelling agent caused a 100% increase in ionic conductivity and major improvement of mechanical strength [426]. The electrolyte was tested successfully in an Al/air battery.

#### 3.1.7. Further Battery Chemistries

For a tin–zinc/graphite battery, a solid composite electrolyte composed of magnesium silicate and sodium phosphate pressed into a PET cloth has been described [427].

For a fluoride-ion battery, a composite electrolyte composed of NH_4_HF_2_, PEO, and *β*-PbSnF_4_ has been prepared [428]. It was tested in a cell with a positive CuF_2_ and a negative Pb/PbF_2_ electrode for up to 50 cycles.

A solid composite electrolyte of PVA cellulose acetate with ammonium triflate as a salt and nano-Al_2_O_3_ as an inorganic filler for a proton battery with σ_RT_ = 2.012 × 10^−3^ S/cm and *t*_+_ = 0.9684 has been reported [429]. Without filler, σ_RT_ = 2.93 × 10^−4^ S/cm was obtained.

A mixed salt of AgI and AgCl was composited with Al_2_O_3_, yielding a solid electrolyte for a silver/I_2_ battery, with the highest ionic conductivity σ_27°C_ = 9.2 × 10^−4^ S/cm at a fraction of 0.3 Al_2_O_3_ [430]. These researchers obtained similar results with another filler, where the highest conduction σ_RT_ = 1.5 × 10^−3^ S/cm was observed at a fraction of 0.2 Fe_2_O_3_ [431]. The reason for the higher effect of Fe_2_O_3_ as compared to Al_2_O_3_ was not specified.

#### 3.1.8. General Aspects and Miscellaneous Observations

The viscosity and dynamics of a mixture (called a composite by the authors) of pectin and an ionic liquid BMIMPF_6_ considered as a possible electrolyte for batteries have been studied with extensive simulations [432]. A composite (actually, following the proposed narrower definition of a composite above, it is a mixture) of PEO and E8 nematic liquid crystals has been studied as a possible electrolyte for solid-state batteries [433]. Properties of mixtures of PEO and variable fractions of the nematic liquid crystal E8 have been investigated, and no charge carriers were identified [434]. Although clearly no DC conductivities were measured, a result of σ_RT_ = 4.36 × 10^−9^ S/cm apparently derived from AC impedance measurements for the most conductive composition was stated. Why this should lead the researchers to recommend the described materials for application in batteries remains blurred. Even with 10 wt.% added NaIO_4_ the resulting value of σ_RT_ = 1.05 × 10^−7^ appears not very attractive [435].

Composite electrolytes based on polyurethane with silica for application in MEMS have been reported [436]. In a general study of blends of PEO and PVP (see Figure 13) with added inorganic fillers ZnO and TiO_2_, evidence was found suggesting the use of some of the studied combinations in energy storage devices [437]. A composite electrolyte of PEO (50 parts) and AgCF_3_SO_3_ (1 part) with 2 wt.% SnO_2_ as the inorganic filler and various amounts of ethylene carbonate as the plasticizer has been prepared and studied [438]. Without plasticizer, σ_RT_ = 3.1 × 10^−6^ S/cm was found, and upon addition, the value went up to 5.4 × 10^−5^ S/cm; at 30 wt.% added liquid with growing plasticizer fraction.

Nanofibers of ceramic NASICON Li_1.5_Al_0.5_Ge_1.5_(PO_4_)_3_ were prepared by electrospinning and composited with PEO, and the composite electrolyte had double the ionic conductivity of the plain PEO [439]. An electrolyte of PVA with 5 wt.% KBr (why the salt is called a composite electrolyte remains unclear) showed an increased ionic conductivity of σ_?_ = 6.24 × 10^−2^ S/cm after electron irradiation. Whether the mentioned temperature applies to the conductivity measurement or the electron irradiation remains unclear. In a high-throughput study of more than 700 ceramics derived from Na_2_ZnSiO_4_-based compounds soaked with an electrolyte solution of NaTFSI in PYR_14_TFSI, relatively high ionic conductivities were observed, but unfortunately, all materials were highly incompatible with sodium commonly used in sodium batteries [440].

Filling mesoporous silica with an ionic liquid EMIMBF_4_ (Figure 23) and combining the product with PEO to form a composite electrolyte yielded a 3-fold increase in ionic conductivity when only 5 wt.% of the IL was incorporated [441]. This increase was attributed to fast ion movement inside the IL-filled pores and channels. The ionic conductivity of a composite of PVdF-HFP and an IL increased by an order of magnitude upon addition of organic solvents like propylene carbonate as a plasticizer [442]. This increase has been attributed to an increase in the concentration of free ions and their higher mobility because of the decreased viscosity of the medium.

Details of modeling polymer/ceramic composite electrolytes at the molecular level have been discussed, with particular attention to the ceramic/polymer interface as the assumed main contributor affecting overall ionic conductivity [443]. Mathematical models have been applied, working towards the calculation of effective conductivities of particular polymer composite electrolytes [444]. Computational prediction methods for dielectric properties relevant for behavior of these materials as electrolytes have been discussed [445]. Computational tools for the development of soft solid composite electrolytes with enhanced ionic conductivities have been presented [446]. Results of a theoretical study of PVA/cellulose composite electrolytes are available [447].

Phase-change materials suitable both as a solid electrolyte and as heat storage materials have been reviewed [448].

A composite electrolyte of Sn_0.9_In_0.1_P_2_O_7_ combined with either PTFE or sulfonated polystyrene-b-poly(ethylene/butylene)-b-polystyrene for a device called a “rechargeable fuel cell”, essentially a cell able to run as an electrolyzer or a fuel cell, has been reported [449].

Composite materials have been applied also in redox flow batteries [450,451].

### 3.2. Elevated Temperature Systems

In batteries employing molten metals as well as other molten electrode materials (e.g., sulfur or metal halides), solid electrolytes have been essential prerequisites to consider such systems. Even in the very specific case of the all-liquid metal accumulator [452] with a molten salt as an electrolyte, advances with solid-ion conductors have attracted attention because their use may avoid some difficulties encountered with molten salt electrolytes. The essential role of solid electrolytes as well as their inherent drawbacks and limitations sketched above have attracted considerable research efforts in this field before the focus has shifted to their use in ambient temperature systems as a replacement of liquid electrolyte (solutions).

Composite solid electrolytes for HT sodium batteries of Na-β″-alumina and YSZ have been prepared by vapor-phase synthesis [453]. Conductivity was σ_RT_ = 1.6 × 10^−3^ S/cm and σ_300°C_ = 1.3 × 10^−1^ S/cm with a fully converted (i.e., α-Alumina + YSZ composite into Na-β″AY) material. Improved mechanical properties of the composite β″-Al_2_O_3_-ZrO_2_ as compared to plain β″-Al_2_O_3_ have been reported [454]. Improved conduction behavior of a two-layer solid electrolyte comprising a dense support of β″-Al_2_O_3_ has been stated [455]. A β″-Al_2_O_3_ –glass composite electrolyte with a resistance of about 20 Ω·cm at 250 °C chemically stable vs. sodium has been described [60].

A composite of gadolinium-doped ceria/magnesia with an optimized composition has been developed for intermediate-temperature solid oxide fuel cells [456]. A composite electrolyte of Ce_0.85_Gd_0.15_O_2_ (CGO) and Sc_2_O_3_-doped ZrO_2_ (ScSZ) for an SOFC has been prepared, where the cell performance with a simple single-layer CGO electrolyte was increased substantially in terms of open circuit voltage and peak power density when a second layer of ScSZ was added [457].

## 4. Conclusions

Given the reported examples of various added components finally yielding a wide range of composite electrolytes for metal and metal-ion batteries with widely varying properties of the negative electrode, several tasks and beneficial effects of these additions can be discerned, which are arranged in the order of importance and relevance—from the author’s perspective:

- Enhanced ionic conductivity;
- Increased transference number of the ion (mostly the cation) of interest;
- Stronger adherence to electrode(s);
- Better inhibition of dendrite formation at the negative electrode;
- Better water retention for hydrogels;
- Higher thermal and mechanical stability;
- Self-healing.

Achieved specific conductivities vary widely; sometimes acceptable values are reached only at temperatures so high above room temperature that practical application appears to be unlikely. Actually, contributions of an electrolyte to the internal cell resistance depend on the thickness of the electrolyte or electrolyte and separator and on properties of the established electrolyte/electrode interface. The latter value is more relevant than a specific value but more difficult to specify for a given cell. At least in correctly evaluated impedance measurements, the Ohmic component of the impedance at *f* → ∞ Hz should provide a value that can even be converted into an aerial one when cell and electrode dimensions are well-defined. The general wish to achieve higher specific values is certainly dominant and appropriate, but even more attractive is a value of the latter resistance that is as small as possible. Reporting such data in future communications would be highly helpful. Equally important but not at all always reported is the cell performance—in particular, cell behavior as a function of current/current density. This numerical value will implicitly allow researchers to report about the establishment of an adequate electrolyte/electrode interface. In particular, with porous electrodes—and most of the positive electrodes are of this type—the establishment of an intimate contact between is a challenge, which goes way beyond the approach sometimes encountered to “just apply a high enough mechanical pressure”.

To determine whether the generalization “better a stable long-term performance at slightly higher internal resistance than a low-resistance cell failing after ten cycles” is acceptable, there is a definite need to test the cell performance for higher cycle numbers. This means, for secondary batteries in portable applications (e.g., mobile phones), 500 to 1000 (Chinese standard GB/T 18287 ≥ 400, with an EU directive aiming at 2000); for cars (standard GB/T 31484), ≥1000; and for stationary storage (Chinese standard GB/T 36276), ≥6000.

## Data Availability

No new data were created or analyzed in this study.
